# Association of whole blood heavy metal concentrations with kidney function

**DOI:** 10.1038/s41598-025-93548-7

**Published:** 2025-03-11

**Authors:** Jiao Zuo, Katrin Huesker, Yvonne Liu, Johann-Georg Hocher, Xiaoli Zhang, Volker von Baehr, Bernhard K. Krämer, Berthold Hocher

**Affiliations:** 1 Department of Nephrology, Charité Universitätsmedizin Berlin, Berlin, Germany; 2 Fifth Department of Medicine (Nephrology/Endocrinology/Rheumatology, Pneumology), University Medical Center Mannheim, University of Heidelberg, Mannheim, Germany; 3 Institute of Medical Diagnostics, IMD, Berlin, Germany; 4 Institute of Pharmacy, Freie Universität Berlin, Berlin, Germany; 5 Reproductive and Genetic Hospital of CITIC-Xiangya, Changsha, China; 6 School of Medicine, Central South University, Changsha, China

**Keywords:** Arsenic, Lead, Mercury, Safety threshold, Kidney function, Environmental sciences, Natural hazards, Diseases, Risk factors

## Abstract

Relatively elevated concentrations of arsenic, lead, and mercury are toxic to the kidneys. However, it is unknown whether kidney function is influenced by these metals in the general population without kidney diseases and without known exposure to these metals. We did a retrospective analysis of data collected from 58,864 outpatients in Germany from January 2014 to October 2022 undergoing measurements of arsenic, lead, and mercury. Routine clinical laboratory parameters were entered into the database if they were analyzed in the same patient within +/- four weeks of the metal analysis. The estimated glomerular filter rate (eGFR) was calculated using the 2021 CKD-EPI equation. The mean age of the study participants was 50.3 ± 17.1, of which 61.8% were women. Complete blood count, CRP, fasting glucose, liver and lipid values, and thyroid function parameters were within the normal range. Median (IQR) eGFR level was 92.14 (79.44-103.85) mL/min/1.73m^2^. Median (IQR) whole blood values for arsenic were 0.8 (0.4–1.5) µg/l, median (IQR) level for lead was 13.6 (9.5–19.5) µg/l, median (IQR) values for mercury were 0.8 (0.3–1.5) µg/l in whole blood. Arsenic (*r*= -0.131, *p* < 0.001, *N* = 11,211), lead (*r* = 0.318, *p* < 0.001, *N* = 21,733), and mercury (*r*= -0.149, *p* < 0.001, *N* = 22,670) levels correlate all inversely with eGFR. When plotting eGFR against whole blood metal concentrations, no lower safety thresholds were found. Multivariate analysis, considering age, sex, CRP, and fasting glucose as confounding factors, confirmed findings of independent associations of arsenic, lead, and mercury on eGFR. Subgroup analysis revealed that this inverse relationship was particularly pronounced in the lowest age tertile of all study participants. Concentrations of arsenic, lead, and mercury correlated independently inversely with eGFR in a German cohort that largely had a normal kidney function with no known exposure to heavy metals.

**Supplementary Information:**

The online version contains supplementary material available at 10.1038/s41598-025-93548-7.

## Introduction

Metals such as arsenic (As), lead (Pb), and mercury (Hg) are widespread in the environment, but have no known biological function in the human body^1^. These metals can enter the body through ingestion, water consumption, inhalation, and/or skin contact^2,3^. In the list of hazardous substances of the Agency for Toxic Substances and Disease Registry (ATSDR), arsenic (As), lead (Pb), and mercury (Hg) are listed as three non-essential metals that are toxic to humans and can cause damage to health even in low concentrations^4^. At high levels, these metals have been shown to be nephrotoxic^5^. Due to their long half-lives, prolonged low-level exposure can lead to accumulation, particularly in the kidneys because they are the main organ for the excretion of heavy metals^6^.

Arsenic is a naturally occurring toxic metalloid that is widely found in the earth’s crust, as well as in drinking water in affected regions. Ground water contamination and the resulting accumulation of arsenic for example in rice is the main source of exposure to naturally occurring inorganic As. Because arsenic is excreted in urine, it can accumulate in the body under conditions of renal insufficiency or failure.

The kidneys are also affected by Pb toxicity, as they are the main route for Pb excretion in humans. This toxicity is mainly caused by Pb-induced lipid peroxidation^7^. Pb further accumulates when the kidney function is impaired, which in turn increases its nephrotoxic effect^8^.

Hg is ubiquitous in nature and occurs in three forms: elemental mercury, organic mercury such as methylmercury and ethyl-Hg, and inorganic Hg^9^. The main concern about all forms of Hg are the adverse effects on kidneys, since they are the natural route of excretion.

In studies of patients with impaired renal function, an increased concentration of these metals in the blood is expected due to the reduced excretion of As, Pb, and Hg. Such studies are therefore unsuitable for making statements about the nephrotoxicity of these metals for patients with no preexising kidney damage.

Whether low concentrations of As, Pb and Hg impair renal function in the general population, however, has not yet been adequately investigated^10,11^. It is therefore still unclear whether there is a lower safe threshold for the blood concentration of As, Pb, and Hg that has no negative effect on kidney function. However, this is essential to find out from a preventive medical perspective, as it must be assumed that everyone is exposed to these metals, and we currently do not know at what concentration they begin to impair kidney function.

## Results

### Characteristics of the study population

**Fig. 1:**
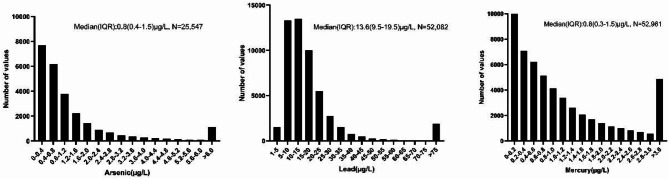
Distribution of the three metals (arsenic, lead, and mercury).

**Fig. 2:**
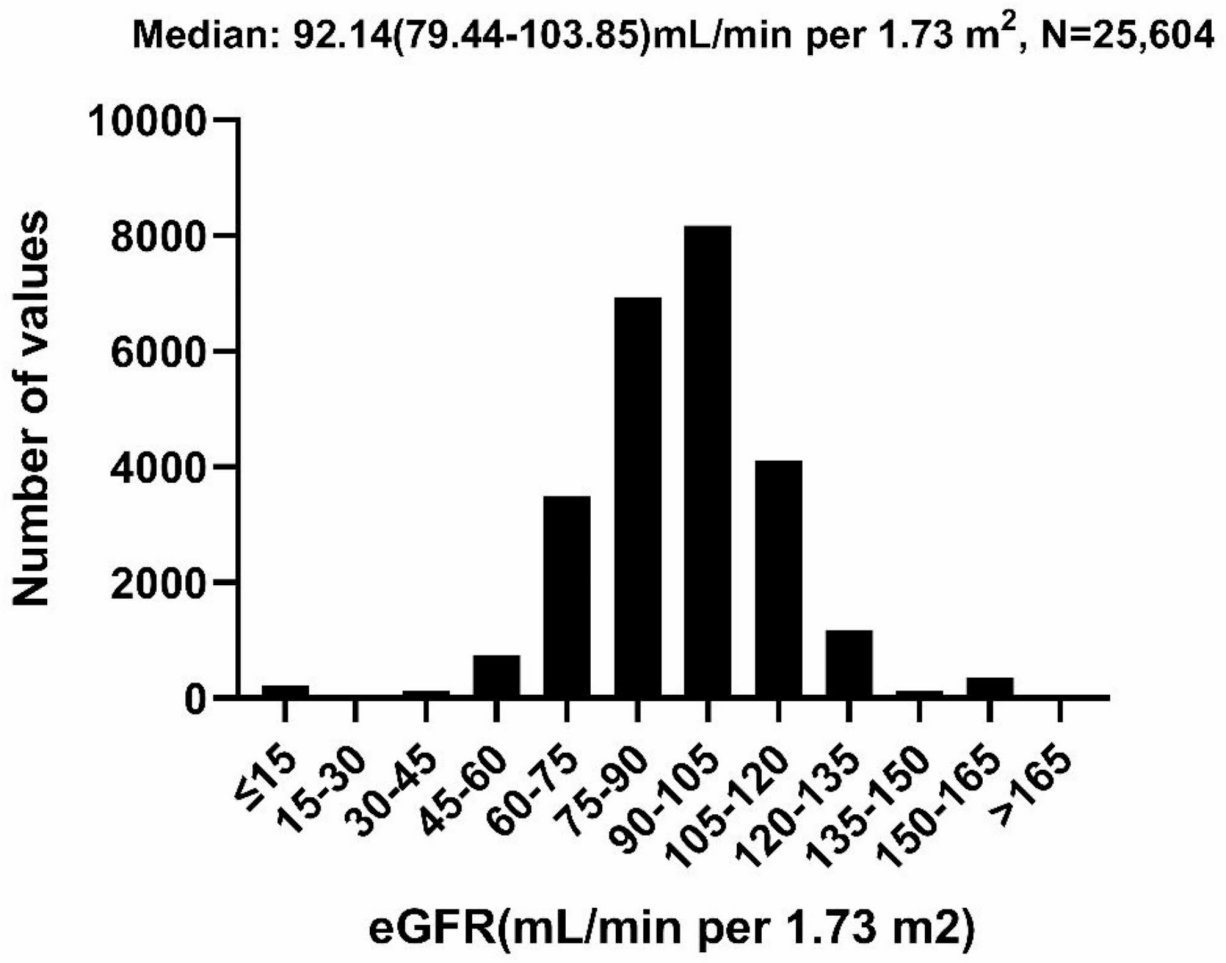
Distribution of eGFR.

In our study, we included 58,864 outpatients. Most of them had no known kidney disease nor heavy metal exposure. There were 22,267 (37.8%) men, 36,569 (62.1%) women, and 28 (< 0.01%) patients with unknown sex. The average age was 50.78 years. Data was available for 25,547 outpatients on arsenic levels (median: 0.8 µg/L), 52,082 outpatients on lead levels (median 13.6 µg/L), and 52,961 outpatients on mercury levels (median: 0.8 µg/L) (Table 1; Fig. 1). The detection limits of the ICP-MS system were sufficiently sensitive to identify traces of arsenic, lead, and mercury in whole blood samples. The lower limits of quantification (LLOQ) for our assays were as follows: arsenic: 0.2 µg/L (7.2% of samples in the current study); lead: 2 µg/L (0.1% of samples in the current study); mercury: 0.2 µg/L (12.5% of samples in the current study). The median (IQR) eGFR level was 92.14 (79.44-103.85) mL/min/1.73m^2^ (Fig. 2).

**Table 1:** Characteristics of the study population.

	*N*	Mean	Medium	SD
Age	58,864	50.78	52.32	16.72
Sex (Male/Female/Unknown)	22,267/36,569/28			
Creatinine (mg/dL)	25,604	0.93	0.84	0.75
CRP (mg/L)	31,430	2.82	0.85	7.75
Basophilic granulocytes (%)	28,558	0.76	0.70	0.35
Eosinophilic granulocytes (%)	28,558	2.67	2.2	2.15
Erythrocytes (million/µl)	37,199	4.56	4.54	0.47
Immature granulocytes (%)	28,539	0.30	0.3	0.21
Leukocytes (1000/µL)	37,066	6.29	5.9	5.62
Lymphocytes (%)	28,556	30.78	30.6	8.28
Hemoglobin (g/dL)	37,185	13.79	13.7	1.39
Hematocrit (%)	37,138	40.62	40.5	4.089
MCH (Hb/number of erythrocytes) (pg)	37,199	30.31	30.4	1.77
MCV (Hk/number of erythrocytes) (fl.)	37,199	89.30	89.1	5.34
MCHC (Hb/HK) (g/dL)	37,199	33.97	34.1	1.46
RDW (erythrocyte volume range) (%)	37,170	13.07	12.9	1.11
Monocytes (%)	28,557	8.35	8.1	2.11
Neutrophilic granulocytes (%)	28,557	57.20	57.3	9.25
Thrombocytes (1000/µL)	37,192	250.02	245	63.50
Alkaline phosphatase (U/L)	19,755	72.22	65	44.88
AST (U/L)	25,480	26.78	24	14.58
ALT (U/L)	25,444	27.24	23	19.59
Glucose (mg/dL)	16,892	102.41	99	20.58
HbA1c (mmol/mol)	17,381	36.58	36	6.47
free T4 (pg/mL)	17,037	11.67	11.5	2.61
TSH (mU/L)	26,183	1.769	1.47	2.374

### Distribution of heavy metals (arsenic, lead, mercury) in different age and sex subgroups

**Fig. 3:**
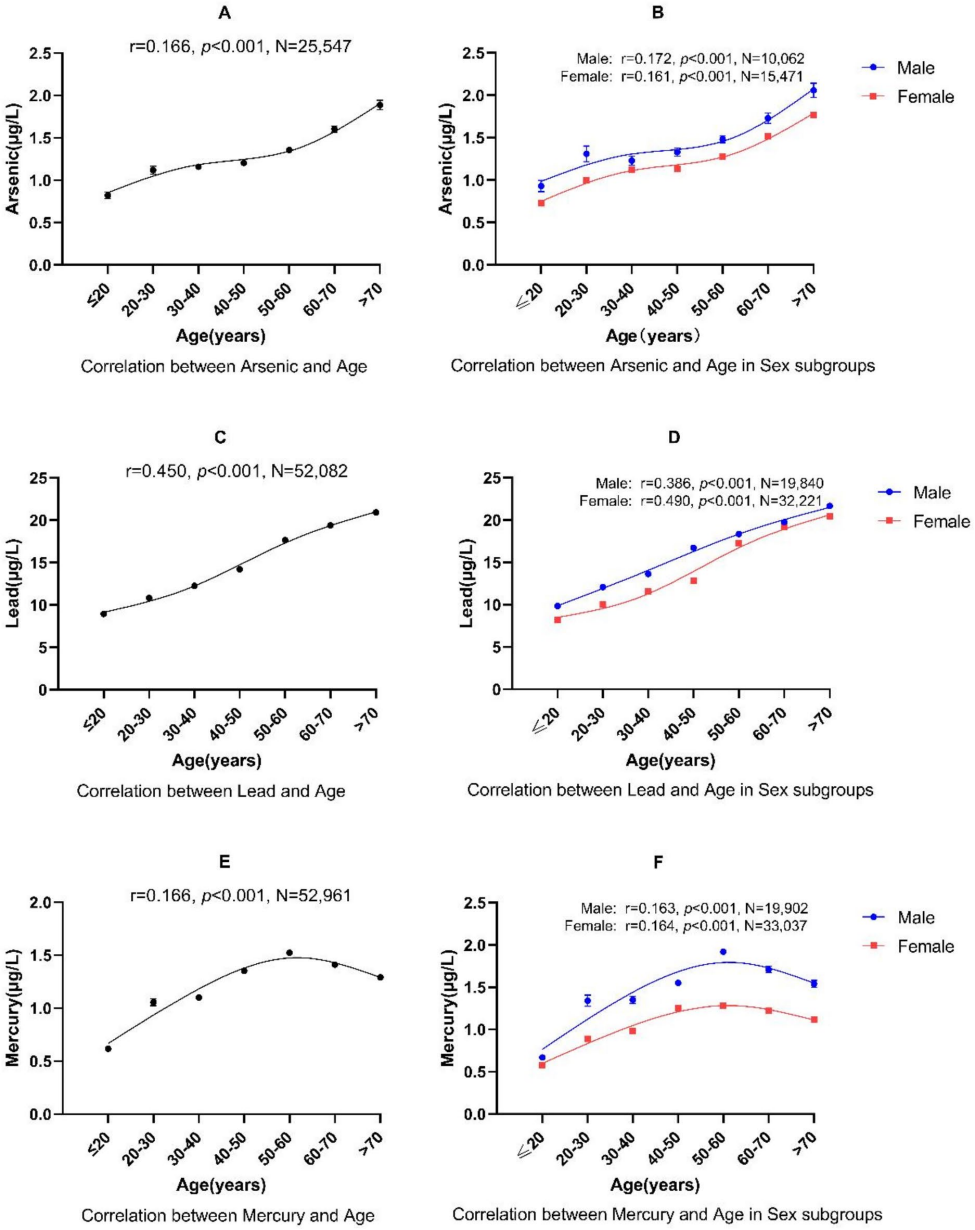
Correlation between metals and age, also considering sex subgroups. Each data point represents mean +/- SEM.

Figure 1 displays, for arsenic, lead, and mercury, that as their whole blood concentration increases, the frequency mostly decreases. Comparing age subgroups (≤ 20, 20s, 30s, 40s, 50s, 60s, and > 70 years old), arsenic levels increase significantly with age (*r* = 0.166, *p* < 0.001, *N* = 25,547) (Fig. 3A). A similar relationship can be seen between age and lead level (*r* = 0.450, *p* < 0.001, *N* = 52,082) (Fig. 3C). Although mercury levels also correlate with age (*r* = 0.166, *p* < 0.001, *N* = 52,961), the increase stops at 50–60 years and even decreases slightly in older age groups (Fig. 3E).

In the age subgroup analysis, all three metals correlated with age in the 40–50 and 50–60 subgroups; lead was associated with age in all age subgroups, except in the 20-30-year-old group; mercury was not associated with age in the 60–70 and > 70 age subgroups (Table 5). In the sex subgroups, Spearman’s correlation showed that arsenic (males: *r* = 0.172, *p* < 0.001, *N* = 10,062; females: *r* = 0.161, *p* < 0.001, *N* = 15,471), lead (males: *r* = 0.386, *p* < 0.001, *N* = 19,840; females: *r* = 0.490, *p* < 0.001, *N* = 32,221), and mercury (males: *r* = 0.163, *p* < 0.001, *N* = 19,902; females: *r* = 0.164, *p* < 0.001, *N* = 33,037) significantly correlate with age in both men and women (Fig. 3B, D, F). Because there were only 28 outpatients of unknown sex (Table 1) and not all data on metals and eGFR was available, these patients were excluded from this correlation analysis.

### Correlation of arsenic, lead, and mercury with eGFR

**Fig. 4:**
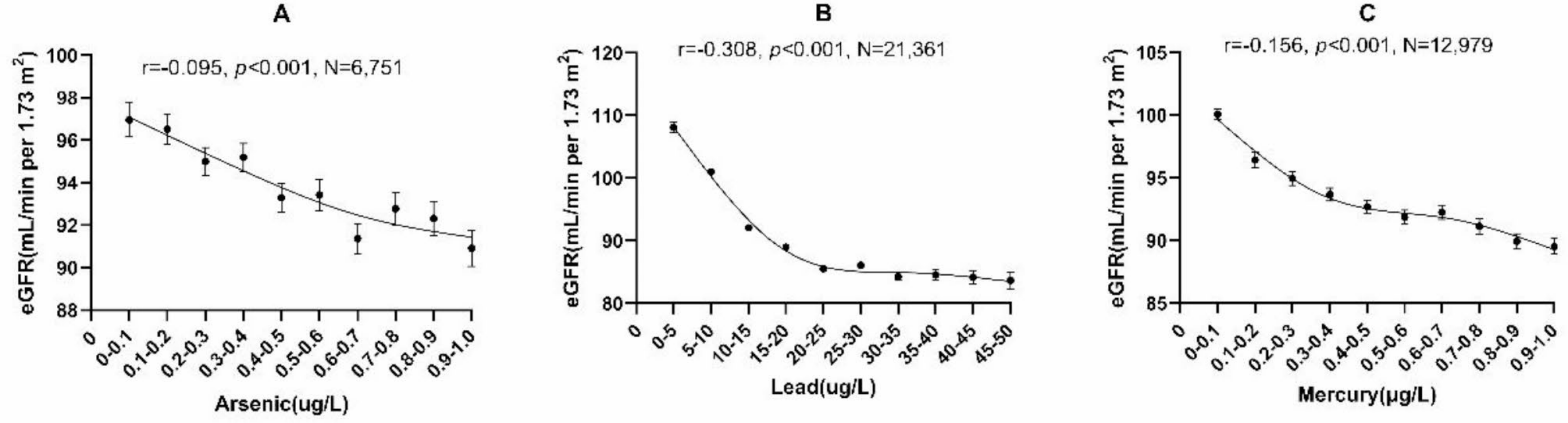
Correlation between metals (low concentration range) and eGFR. Metal concentrations were grouped into ten different metal concentration groups: Arsenic and Mercury: 0-0.1; 0.1–0.2; 0.2–0.3; 0.3–0.4; 0.4–0.5; 0.5–0.6; 0.6–0.7; 0.7–0.8; 0.8–0.9; 0.9–1.0 µg/L. Lead: 0–5; 5–10; 10–15; 15–20; 20–25; 25–30; 30–35; 35–40; 40–45; 45–50 µg/L. Each data point represents mean +/- SEM.

Supplementary Fig. 1 showed that arsenic (*r*= -0.131, *p* < 0.001, *N* = 11,211), lead (*r*= -0.318, *p* < 0.001, *N* = 21,733), and mercury (*r*= -0.149, *p* < 0.001, *N* = 22,670) all inversely correlate with eGFR in our study. With an increasing arsenic concentration, eGFR decreased (Fig. 4); up to a concentration of about 0.8–1.0 µg/L. At higher arsenic concentrations, eGFR plateaued. A similar trend was seen for lead and mercury – at a certain lead and mercury concentration eGFR also did not fall further (Fig. 4). Multivariate linear regression showed that arsenic (unstandardized coefficient B: -0.224, *p* = 0.043), lead (unstandardized coefficient B: -0.031, *p* = 0.025), and mercury (unstandardized coefficients B: -0.392, *p* < 0.001) correlated inversely with eGFR independently of the confounding factors age, sex, CRP, and fasting glucose (Tables 2, 3 and 4). Multivariate linear regression analysis in the subgroups of patients with eGFR above 60 mL/min for arsenic and mercury are significantly correlated with eGFR (Supplementary Tables 1–6).

**Table 2:** Multivariate linear Regression – Arsenic (Dependent variable: eGFR).

Variables	Multivariate Linear Regression	
	β Coefficient / 95%CI	*p* Value
Constant	140.762 (137.428 ~ 144.096)	< 0.001
Age	-0.751 (-0.780 ~ 0.722)	< 0.001
Sex	-1.656 (-2.597~-0.714)	< 0.001
CRP	-0.033 (-0.085~-0.020)	0.220
Glucose	-0.064 (-0.086 ~ 0.041)	< 0.001
Arsenic	-0.224 (-0.442~-0.007)	0.043

**Table 3:** Multivariate linear Regression – Lead (Dependent variable: eGFR).

Variables	Multivariate Linear Regression	
	β Coefficient / 95%CI	*p* Value
Constant	139.716 (137.366 ~ 142.067)	< 0.001
Age	-0.779 (-0.800~-0.758)	< 0.001
Sex	-1.151 (-1.827~-0475)	< 0.001
CRP	-0.047 (-0.083~-0.011)	0.010
Glucose	-0.049 (-0.065~-0.034)	< 0.001
Lead	-0.031 (-0.058~-0.004)	0.025

**Table 4:** Multivariate linear Regression – Mercury (Dependent variable: eGFR).

Variables	Multivariate Linear Regression	
	β Coefficient / 95%CI	*p* Value
Constant	140.184 (137.870 ~ 142.498)	< 0.001
Age	-0.782 (-0.802~-0.763)	< 0.001
Sex	-1.375 (-2.035~-0.715)	< 0.001
CRP	-0.044 (-0.080~-0.008)	0.016
Glucose	-0.050 (-0.065 ~ 0.034)	< 0.001
Mercury	-0.258 (-0.392~-0.125)	< 0.001

### Interaction of the effects of arsenic, lead, and mercury on kidney function

To examine the potential interaction effects of heavy metals on kidney function, we categorized the concentrations of each metal into two groups based on the median value: one group with concentrations below the median (Low) and another with concentrations above the median (Elevated). We then analysed the interactions between these groups (Table 5).

**Table 5:** Spearman-correlation between eGFR and metal concentrations in different age groups.

	Arsenic			Lead			Mercury		
Age	r	p	N	r	P	N	r	p	N
≤ 20	-0.027	0.540	535	0.132**	< 0.001	1103	-0.113**	< 0.001	1102
20–30	-0.223**	< 0.001	625	-0.051	0.081	1179	-0.193**	< 0.001	1255
30–40	-0.031	0.320	1035	-0.088**	< 0.001	2157	-0.092**	< 0.001	2454
40–50	-0.092**	< 0.001	2050	-0.084**	< 0.001	4070	-0.102**	< 0.001	4318
50–60	-0.025	0.164	3222	-0.060**	< 0.001	6049	0.188**	< 0.001	6240
60–70	-0.026	0.227	2146	-0.046**	0.004	4036	-0.019	0.233	4159
> 70	-0.052**	0.037	1595	-0.068**	< 0.001	3131	-0.018	0.322	3134

**Fig. 5:**
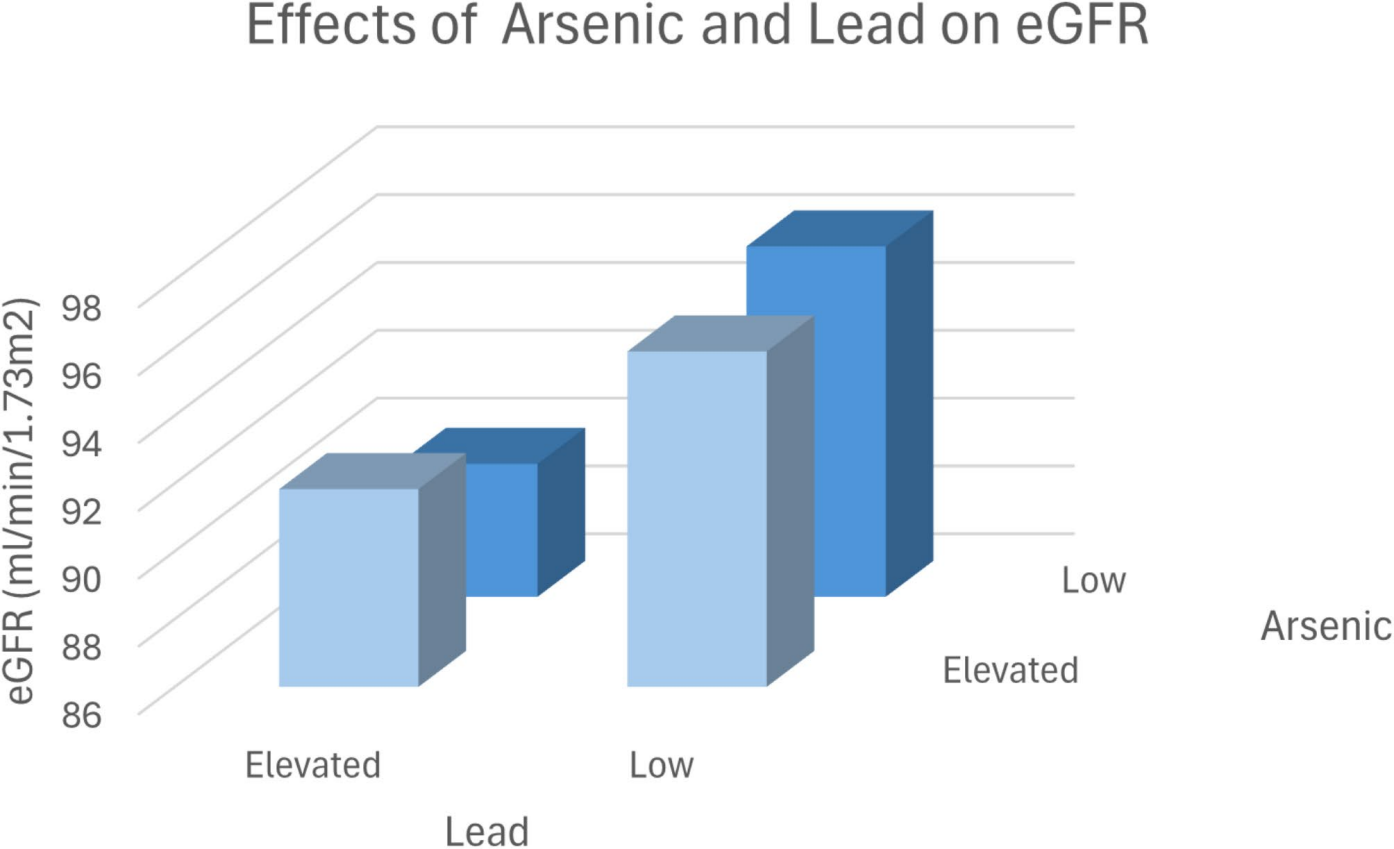
3D Plot-Effects of Arsenic and Lead on eGFR. The patients were divided into four groups according to the median of the metal concentrations: Group 1: Low Lead/Low Arsenic: eGFR ± SEM: 96.33 ± 0.158 ml/min/1.73m^2^, *N* = 15,832. Group 2: Low Lead/ Elevated Arsenic: eGFR ± SEM: 95.89 ± 0.124 ml/min/1.73m^2^, *N* = 25,670. Group 3: Elevated Lead/Low Arsenic: eGFR ± SEM: 89.92 ± 0.141 ml/min/1.73m^2^, *N* = 17,339. Group 4: Elevated Lead/ Elevated Arsenic; eGFR ± SEM: 91.83 ± 0.116 ml/min/1.73m^2^, *N* = 27,177. Group 1 versus group 4: *p* < 0.001. Group 2 versus group 4: *p* < 0.001. Group 3 versus group 4: *p* = 0.017.

Figure 5 shows the effects of arsenic and lead on eGFR.

**Fig. 6:**
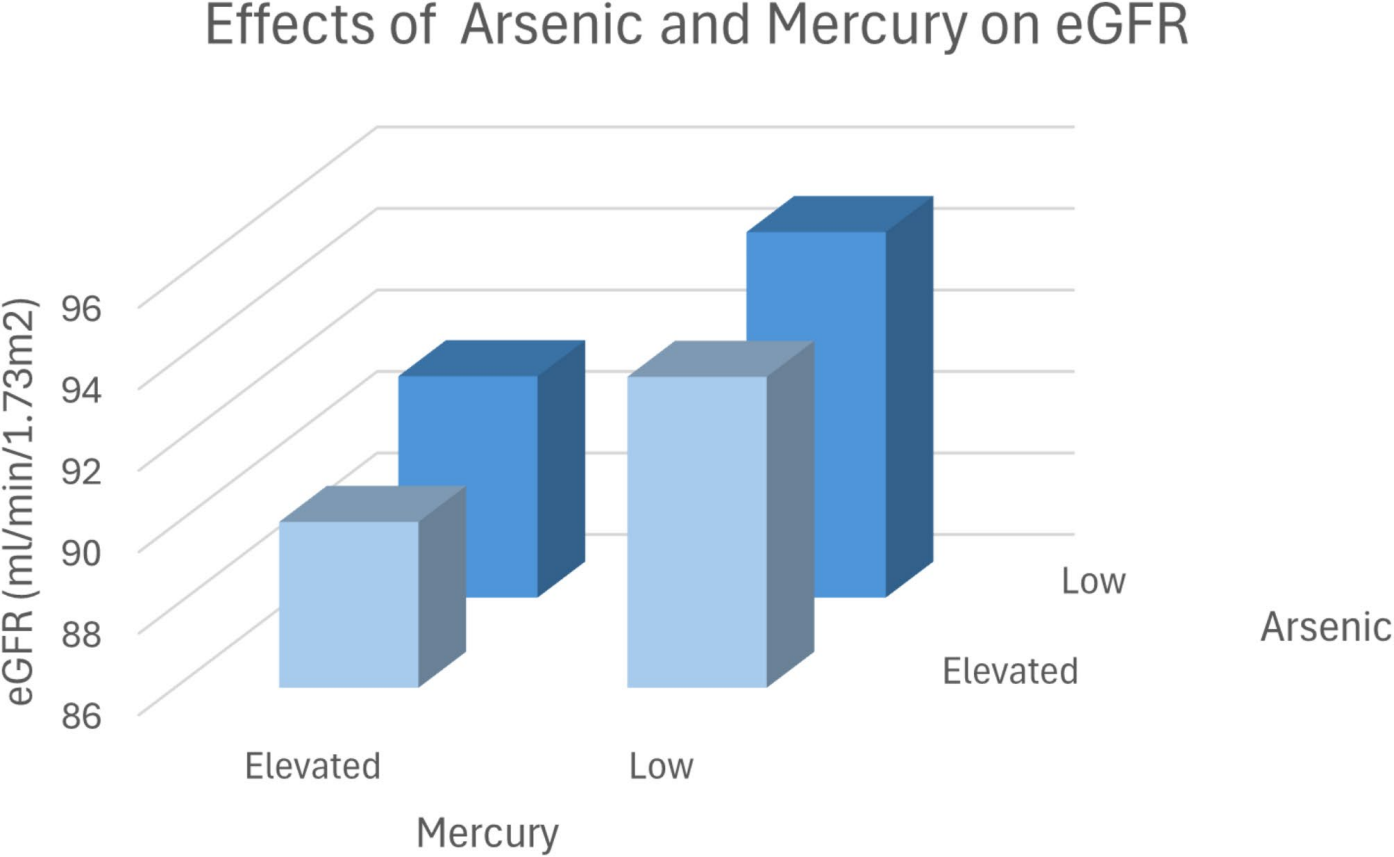
3D Plot-Effects of Arsenic and Mercury on eGFR. The patients were divided into four groups according to the median of the metal concentrations: Group 1: Low Arsenic/Low Mercury: eGFR ± SEM: 94.97 ± 0.158 ml/min/1.73m^2^, *N* = 16,650. Group 2: Low Arsenic / Elevated Mercury: eGFR ± SEM: 91.43 ± 0.138 ml/min/1.73m^2^, *N* = 17,182. Group 3: Elevated Arsenic /Low Mercury: eGFR ± SEM: 93.63 ± 0.157 ml/min/1.73m^2^, *N* = 16,376. Group 4: Elevated Arsenic / Elevated Mercury; eGFR ± SEM: 90.07 ± 0.134 ml/min/1.73m^2^, *N* = 16,908. Group 1 versus group 4: *p* < 0.001. Group 2 versus group 4: *p* < 0.001. Group 3 versus group 4: *p* < 0.001.

**Fig. 7:**
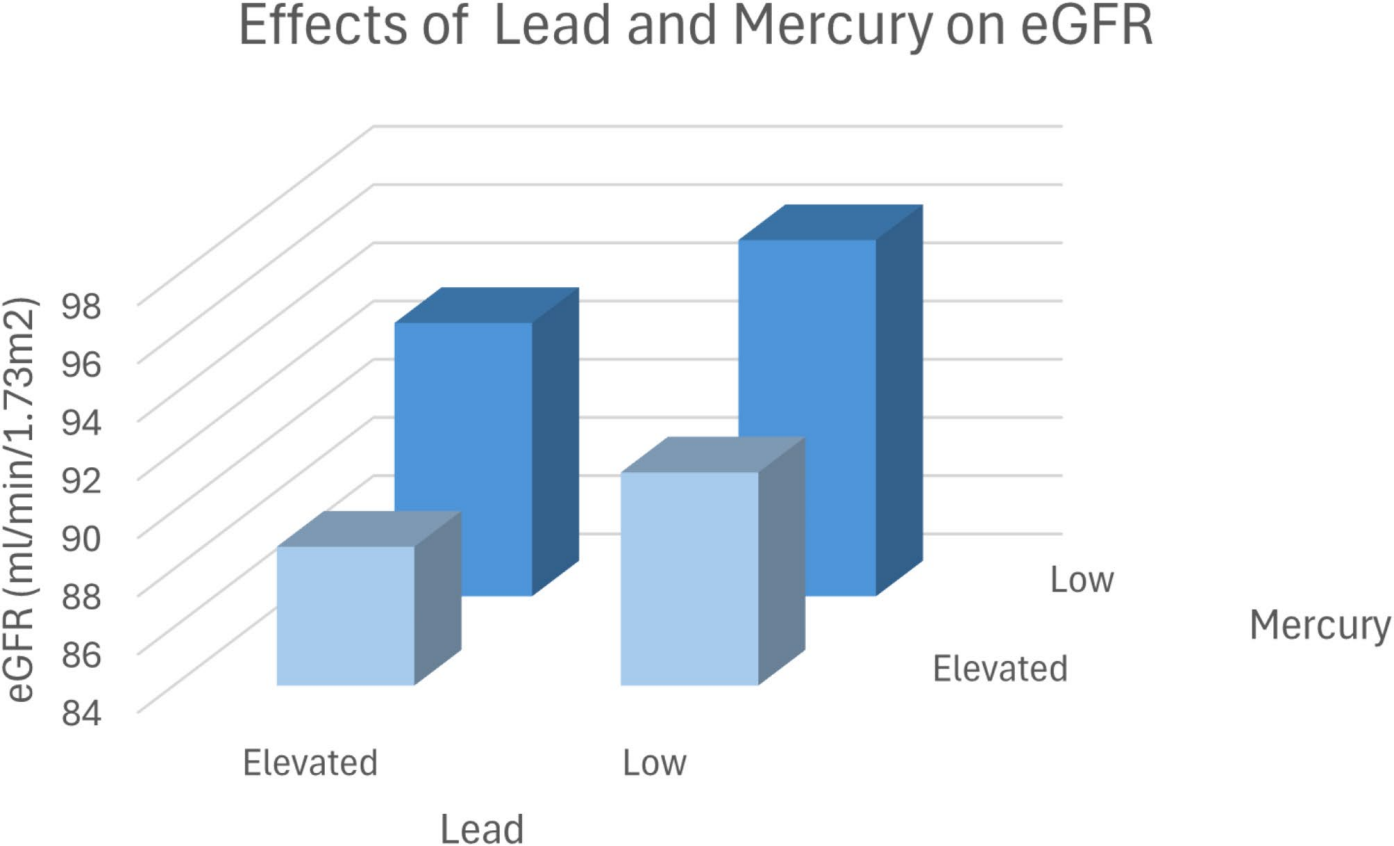
3D Plot-Effects of Lead and Mercury on eGFR. The patients were divided into four groups according to the median of the metal concentrations: Group 1: Low Mercury /Low Lead: eGFR ± SEM: eGFR: 96.24 ± 0.142 ml/min/1.73m^2^, *N* = 21,042. Group 2: Low Mercury / Elevated Lead: eGFR ± SEM: eGFR: 91.32 ± 0.131 ml/min/1.73m^2^, *N* = 22,549. Group 3: Elevated Mercury /Low Lead: eGFR ± SEM: eGFR: 93.39 ± 0.130 ml/min/1.73m^2^, *N* = 21,574. Group 4: Elevated Mercury / Elevated Lead; eGFR ± SEM: eGFR: 88.77 ± 0.115 ml/min/1.73m^2^, *N* = 23,081. Group 1 versus group 4: *p* < 0.001. Group 2 versus group 4: *p* < 0.001. Group 3 versus group 4: *p* < 0.001.

Figure 6 illustrates the effects of arsenic and mercury on eGFR,

Figure 7 demonstrates the effects of lead and mercury on eGFR.

The interaction of metals—arsenic, lead, and mercury—on estimated glomerular filtration rate (eGFR) is notable for its compounding nephrotoxic effects, as demonstrated in the study data from Figs. 5, 6 and 7; Tables 6, 7 and 8. The findings reveal that combined exposure to these metals results in a greater decrease in eGFR compared to exposure to each metal individually, underscoring a potential synergistic toxicity. For instance, Fig. 5 shows that patients with both elevated lead and arsenic levels had a significantly lower eGFR (91.83 ml/min/1.73 m²) compared to those with lower concentrations (96.33 ml/min/1.73 m²), a statistically significant difference (*p* < 0.001). This pattern holds for arsenic-mercury (Fig. 6) and lead-mercury interactions (Fig. 7), suggesting that kidney function diminishes as exposure levels increase.

**Table 6:** Multivariate linear regression –Arsenic and lead interaction (Dependent variable: eGFR).

Variables	Multivariate Linear Regression	
	β Coefficient / 95%CI	*p* Value
Constant	140.24(138.617 ~ 141.864)	< 0.001
Age	-0.76(-0.773~ -0.747)	< 0.001
Sex	-1.217(-1.635~-0.799)	< 0.001
CRP	-0.04(-0.062~ -0.017)	< 0.001
Glucose	-0.056(-0.066~ -0.046)	< 0.001
Arsenic-Lead Interaction	-0.551(-0.896~ -0.205)	0.002

To study the interaction of metals concentrations on eGFR each metal concentrations were divided in a low and an elevated group by the median of the given metal concentration. Next, we created groups of low arsenic and low lead, low arsenic and elevated lead, elevated arsenic and low lead and finally elevated arsenic and elevated lead. These groups were used to test the interaction of the metal on eGFR considering confounding factors such as age, sex and fasting glucose.

**Table 7:** Multivariate linear regression –Arsenic and mercury interaction (Dependent variable: eGFR).

Variables	Multivariate Linear Regression	
	β Coefficient / 95%CI	*p* Value
Constant	141.337 (139.904 ~ 142.769)	< 0.001
Age	-0.772 (-0.784 ~ -0.76)	< 0.001
Sex	-1.406 (-1.787 ~ -1.025)	< 0.001
CRP	-0.04(-0.061 ~ -0.019)	< 0.001
Glucose	-0.055(-0.064~ -0.046)	< 0.001
Arsenic-Mercury Interaction	-0.697 (-0.965~ -0.428)	< 0.001

To study the interaction of metals concentrations on eGFR each metal concentrations were divided in a low and an elevated group by the median of the given metal concentration. Next, we created groups of low arsenic and low mercury, low arsenic and elevated mercury, elevated arsenic and low mercury and finally elevated arsenic and elevated mercury. These groups were used to test the interaction of the metal on eGFR considering confounding factors such as age, sex and fasting glucose.

**Table 8:** Multivariate linear regression –Lead and mercury interaction (Dependent variable: eGFR).

Variables	Multivariate Linear Regression	
	β Coefficient / 95%CI	*p* Value
Constant	139.796 (138.569 ~ 141.023)	< 0.001
Age	-0.764 (-0.774 ~ -0.754)	< 0.001
Sex	-1.033 (-1.362 ~ -0.704)	< 0.001
CRP	-0.047(-0.065 ~ -0.03)	< 0.001
Glucose	-0.051(-0.059~ -0.043)	< 0.001
Lead-Mercury Interaction	-0.777 (-1.013~ -0.542)	< 0.001

To study the interaction of metals concentrations on eGFR each metal concentrations were divided in a low and an elevated group by the median of the given metal concentration. Next, we created groups of low lead and low mercury, low lead and elevated mercury, elevated lead and low mercury and finally elevated lead and elevated mercury. These groups were used to test the interaction of the metal on eGFR considering confounding factors such as age, sex and fasting glucose.

Multivariate regression analysis shows that combined high-level exposure to arsenic and lead (*p* = 0.002), arsenic and mercury (*p* < 0.001), and lead and mercury (*p* < 0.001) independently correlates with a lower eGFR, even after adjusting for confounders such as age, sex, and glucose levels (Tables 6, 7 and 8).

In summary, the combined exposure to these metals appears to exacerbate renal impairment, as evidenced by the decreased eGFR values across different groupings.

Environmental toxins may act on the human body in a sex-dependent manner, which is why we considered sex as a confounding factor in the multivariate analysis and in addition analyzed the relationship between metal concentrations and eGFR in women and men separately. Overall, this analysis revealed no major differences concerning the renal effects of these metals between women and men (Supplementary Fig. 2).

**Fig. 8:**
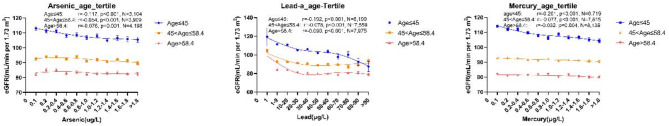
Correlation between metals and eGFR in Tertile Age subgroups. Correlation analysis by tertiles of age of the study participants (age ≤ 45 years old, 45 < Age ≤ 58.4 years old, Age > 58.4 years old) and analyzed by spearman correlation.

Figure 8 shows the correlation of eGFR and toxic metal concentrations in age tertiles of the participants indicating a particularly pronounced inverse relationship in the lowest age tertile. To illustrate the effects of age and sex for the effects of metal concentrations on eGFR, we additional created subgroup analysis (see supplementary Figs. 3–14.): These 3D plots (Supplementary Figs. 3–5, Female Group): illustrate the effects of metal combinations (Arsenic-Lead, Arsenic-Mercury, Lead-Mercury) on eGFR in females. The plots show a decrease in eGFR with elevated levels of each metal combination, highlighting the compounded nephrotoxic effects in female participants.

Analysis of metal combinations with eGFR for male participants are shown in Supplementary Figs. 6–8). Findings were comparable to those seen in female study participants. In Supplementary Figs. 9–11 analysis of metal interactions in older subjects (age above the median of the entire study population) are shown. Supplementary Figs. 12–14 shows similar analysis in participants whose age was below the median age of the study population.

## Discussion

In the present study, we included 58,864 outpatients from 2014 to 2022. This is by far the largest study analyzing the association between blood concentrations of arsenic, lead, and mercury with kidney function in the general population in Central Europe. The impact of arsenic, lead, and mercury – especially mercury – on kidney function has so far been mainly analyzed in Asian populations, however, in smaller studies. The size of the study enabled for the first time an analysis of potential thresholds for toxicity of these metals concerning kidney function.

Arsenic and lead concentrations in whole blood increase throughout life, whereas mercury concentrations increase until middle age and then remain relatively constant. The association of Pb, Hg, and As with eGFR is independent of risk factors such as age, sex, CRP, and fasting glucose in the general German population. Our analysis suggests that a safe lower limit for no effects of Pb, Hg, and As on eGFR could not be detected.

### Age-dependent accumulation of whole blood concentrations of arsenic, lead, and mercury

The kidneys receive a high percentage of blood, about 20% of the cardiac output, and any damage to this filtration barrier can lead to impaired renal function and/or albuminuria. They are one of the main target organs for heavy metal toxicity^12–14^: when the filtration capacity of the glomeruli decreases, fewer ionized heavy metals are filtered by the kidneys, resulting in increased levels of heavy metals in the body. Most of our study participants had a normal eGFR, which is a standard measure of kidney function, considering their age – meaning that there was no evidence of pre-existing kidney disease. It was hence possible to analyze the age-dependent increase of heavy metals in a huge number of patients. This analysis showed that arsenic and lead whole blood concentrations increase throughout life in the general German population. Studies mainly done in Asian populations came to similar conclusions. Exposure to arsenic can occur through contaminated water, food, and air. Chronic exposure leads to its accumulation in the body, increasing concentration in the blood. Studies like those by Navas-Acien et al.^15^ and Rahman et al.^16^ have highlighted the persistence of arsenic in the body, where concentrations often rise with age due to cumulative exposure from various sources. Similarly, exposure to lead, often from sources like paint, water pipes, and certain working environments, also leads to its accumulation in the body over time. Lead accumulates in bones and can be released into the blood during various life events, such as pregnancy or menopause, causing an increase in its blood concentration^17^. In our large study cohort, we rather observed a smooth increase of lead with aging, and an increased lead release from bones in women during menopause was not clearly seen. The current exposure to arsenic and lead in Germany appears to exceed the body’s natural elimination capacity for these metals in subjects with healthy kidneys, resulting in a lifelong increase in the concentration of these metals in the blood.

The accumulation of mercury in whole blood samples is different from the other metals in our study. The concentration of mercury increases continuously until the age range of 50–60 years, after which it remains relatively stable at a high level (Fig. 2E).

Our data concerning age-dependent mercury accumulation in the human body in Germany fits very well with a large study from Japan likewise showing that the accumulation of mercury in the human body can be age-dependent and peaks at around 50 years and slightly decreases thereafter^18^. The primary sources of mercury exposure in Germany include consumption of certain fish species that may contain elevated mercury levels, occupational exposure in industries, and historically, the use of dental amalgam fillings, although their use has declined rapidly in recent years. It remains unclear which of these factors explain the similar age-dependent mercury accumulation pattern in Japan and Germany.

After grouping age in tertiles according to age, (age ≤ 45 years old, 45 < Age ≤ 58.4 years old, Age > 58.4 years old), the correlation between eGFR and metals (arsenic, mercury and lead) in younger subgroups were much stronger than the other two subgroups as seen by the R-value of correlation, see details in Fig. 8. The supplementary Figs. 5–7 and Fig. 8 showed with elevating arsenic, lead, and mercury levels, the eGFR values decrease in both older and younger groups. Although the trend of eGFR reduction with metal exposure persists in younger groups, the impact appears less severe compared to the elderly group, the older individuals are especially vulnerable to renal function impairment from combined metal exposure, suggesting that age is a significant modifier of susceptibility to nephrotoxic metals.

The age-dependent increase of lead, mercury, and arsenic in whole blood sample may accelerate aging in human organs including the kidneys. Among carefully screened healthy kidney donors, GFR declines at a rate of 6.3 ml/min/1.73m^2^ per decade^19^. Another study showed that among apparently healthy adults, there was a linear decline of GFR beyond the age of 30 years, such that by the age of 90 years, GFR was reduced by an average of 46% from that found in the youth^20^. In humans, longitudinal studies show increased oxidative stress in normal aging and CKD^21^. Heavy metals causing oxidative stress besides other toxic effects in the kidneys may accelerate aging of the kidney^22–24^. This hypothesis fits with the observation that with age, eGFR decreases, correlating inversely with heavy metal concentrations^10,25^.

### Interaction of arsenic, lead, and mercury on eGFR

Animal studies prove a causal detrimental impact of lead, arsenic and mercury exposure on kidney function ^26–29^. However, combined effects were not analyzed in animal models. In our study, we observed a significant interaction between arsenic, lead, and mercury and their combined effect on kidney function (Figs. 5, 6 and 7), as measured by the estimated glomerular filtration rate (eGFR). Individually, each of these metals exhibited an inverse relationship with eGFR, consistent with their known nephrotoxic properties. However, our findings suggest that concurrent exposure to multiple metals exacerbates their detrimental impact on kidney function.

The combined exposure to high levels of these metals resulted in a more pronounced reduction in eGFR compared to individual exposures. For example, patients with elevated levels of both lead and arsenic had a significantly lower eGFR than those with high levels of either metal alone. This synergistic effect may be due to the cumulative burden on renal detoxification processes, overwhelming the kidneys’ capacity to excrete these toxic substances.

These interactions underscore the importance of considering cumulative environmental exposures when assessing kidney function and setting safety thresholds. Traditional risk assessments often focus on single-metal exposures, potentially underestimating the risk posed by combined exposures in real-world settings. The little eGFR reduction differences form male and female groups (Supplementary Figs. 3–8) potentially due to sex-specific metabolic and physiological responses to these metals. Our findings highlight the need for revised guidelines that account for the interaction between multiple heavy metals, particularly in populations with chronic exposure.

Although it would be ideal to analyze the interaction of all three metals simultaneously, this was not feasible in our study due to the limited statistical power when dividing the cohort into smaller subgroups. The combination of three metals created subgroups with insufficient sample sizes to detect meaningful interactions, potentially leading to unreliable conclusions. This limitation underscores the challenges in studying complex environmental exposures where multiple toxins interact, necessitating larger cohorts or alternative statistical approaches to fully understand these interactions.

Overall, the data suggest that even low-level exposure to multiple heavy metals can lead to a significant change in eGFR, emphasizing the importance of minimizing environmental and occupational exposure to these toxic elements.

## Limitations and strengths

A major strength of our study is the population size, enabling the analysis of thresholds for the associations of arsenic, lead, and mercury with eGFR and that we have been able to consider some confounders such as age, sex, CRP, and fasting glucose as compared to the other published much smaller studies (see supplementary table). However, our findings are limited by the difficulty in fully adjusting for potential additional confounders, including tobacco use, preexisting hypertension and information about other clinical disease conditions that might contribute to impairment of GFR in the general population. Secondly, we could not differentiate between organic and inorganic forms of toxic metals, such as arsenic and mercury, which have distinct toxicological profiles. Unfortunately, our study was limited to measuring total metal concentrations using ICP-MS, which does not differentiate between organic and inorganic species^28^. This is a limitation, as inorganic forms are generally more toxic. Future research should incorporate speciation analysis to distinguish between these forms, providing a more nuanced understanding of their specific health impacts. Despite this limitation, our findings on total metal concentrations still offer valuable insights into overall exposure and its association with kidney function. Furthermore, toxins are also excreted via faces, which we did not measure because it is unlikely to directly reflect kidney function. Next, our study indicated that the whole blood concentrations of the analyzed toxic metals raise with age. However, this not necessarily reflect metal accumulation in the body, since we do not have any measurement of bone metal concentrations in our study.

Lastly, our study is an association study considering cofounding factors, but it is an association study, causality can not be proven by this study type. However, a placebo-controlled double-blind clinical trial is simply not possible due to ethic reasons, thus this study type used in the current study provides the highest ethically possible level of evidence for our research question.

## Conclusions

Our study analyzed the association between three non-essential metals (arsenic, lead, and mercury) and eGFR in the general population in Germany. It is the largest study focusing on kidney function and these non-essential metals so far, and it is the first European metal study in the general population. In our study, the three metals had significant inverse correlations with eGFR, which were independent of confounding factors for chronic kidney disease. Given our data, it looks that there are no lower safe threshold blood concentrations for As, Pb, and Hg concerning potential harmful effects on kidney function.

## Methods

### Study population

We performed a retrospective analysis of data collected from 58,864 patients getting measurements of at least one of the metals arsenic, lead, and mercury in full blood samples. Data was collected from January 2014 to October 2022. All measurements were done at the Institute for Medical Diagnostics Berlin-Potsdam, Germany (IMD: https://www.imd-berlin.de/en/laboratory.html). Our laboratory is specialized in preventive medicine. Most of the physicians in Germany sending blood to us for analysis do this for preventive medicine reasons. This also explains why in most cases, concentrations of metals were rather low in the whole blood samples, see description of the study population and the description of metal distributions in our cohort.

Besides arsenic, lead, and mercury, we added the following clinical laboratory parameters to the database, if they were measured in the same patient within +/- four weeks of the metal analysis. The following parameters were added: creatinine, C-reactive protein (CRP), basophilic granulocytes, eosinophilic granulocytes, erythrocytes, immature granulocytes, leukocytes, lymphocytes, hemoglobin, hematocrit, mean corpuscular hemoglobin (MCH), mean corpuscular hemoglobin concentration (MCHC), mean corpuscular volume (MCV), red blood cell distribution width (RDW), monocytes, neutrophilic granulocytes, thrombocytes, alkaline phosphatase, glutamic oxaloacetic transaminase (GOT), glutamic pyruvic transaminase (GPT), glucose, glycated hemoglobin (HbA1c), free thyroxine (fT4), thyroid-stimulating hormone (TSH)). The estimated GFR (eGFR) was calculated according to the CKD-EPI 2021 equation: eGFR = 142×min(Scr/κ,1) ^α^ × max(Scr/κ,1)^−1.2000^ × 0.9938^Age^×1.012, where Scr is serum creatinine, κ is 0.7 for females and 0.9 for males, α is -0.241 for females and − 0.302 for males, min indicates the minimum of Scr/κ or 1, and max indicates the maximum of Scr/κ or 1.

This investigation was carried out in compliance with the Declaration of Helsinki Principles for data collection and the ethical requirements of the Institute for Medical Diagnostics Berlin (IMD Berlin), which waived the need for informed permission.

### Measurement of arsenic, lead, and mercury concentration

Metal exposure can be assessed using whole blood ICP-MS measurements or urine analysis^29^. Blood measurements directly correlate with kidney function, reflecting both recent and chronic metal exposure, and are less influenced by hydration, making them more consistent. However, blood sampling is more invasive and may miss high exposure peaks that urine can detect. Urinary analysis is non-invasive and better for identifying acute exposure, but results can vary with hydration and may not directly correlate with kidney damage^29^. Given the study’s focus on chronic exposure and kidney function, whole blood measurements by ICP-MS are more appropriate, despite the advantages of urine analysis for acute exposure monitoring.

Arsenic, lead, and mercury were analyzed by inductively coupled plasma-mass spectrometry (ICP-MS; Thermo Fisher, Bremen, Germany). For sample preparation, whole blood samples were diluted in a high-purity ammonia solution combined with a surfactant to ensure consistent sample flow and prevent matrix effects during analysis. Whole blood samples were diluted 1:20 in high purity 0,1% NH3 (Suprapur, Supelco) / 0,02% Lutrol F88 (Applichem). Subsequent ICP-MS analyses were performed in collision/reaction cell mode, using internal and external standard calibration (Elemental Scientific). We used a Thermo Fisher ICP-MS system, which operates in collision/reaction cell mode to minimize interferences and enhance the accuracy of metal quantification. Internal standards were used to correct for any potential instrument drifts and matrix effects.

Results of ICP-MS measurements represented the total concentrations in whole blood samples of the respective elements, including organic and inorganic species. The ICP-MS system’s detection limits were sufficiently low to detect traces of arsenic, lead, and mercury in full blood samples. The lower limits of quantification (LLOQ) for our assays were:

- Arsenic: 0.2 µg/L.
- Lead: 2 µg/L.
- Mercury: 0.2 µg/L.

In cases where metal concentrations were below the detection limits, we employed a conservative approach to data handling. We assigned a value equal to the midpoint between zero and the LLOQ for these samples to avoid any bias in statistical analysis.

Regular calibration using certified reference materials ensured the accuracy of the measurements. Quality control samples were analyzed alongside study samples to confirm the consistency and reliability of the data.

### Statistical analysis

Data was analyzed using SPSS (Statistical Package for Social Sciences), version 29.0 (IBM Corporation, Armonk, New York, USA) and statistical significance was defined as *p* < 0.05. Figures 1, 2, 3 and 4 were created using GraphPad Prism 6 (GraphPad Software Inc., San Diego, California, USA) and Figs. 5, 6 and 7, supplementary Figs. 3–14 using Excel. Categorical variables were presented as frequency (percentage, %), arsenic, lead and mercury are presented as median (interquartile range, IQR) and continuous variables as mean (standard error of mean, SEM). Spearman correlation was used for univariate analysis. We divided the cohort into ten groups, according to the concentration of metals: arsenic and mercury (0–0.2 µg/L; 0.2–0.4 µg/L; 0.4–0.6 µg/L; 0.6–0.8 µg/L; 0.8–1.0 µg/L; 1.0–1.2 µg/L; 1.2–1.4 µg/L; 1.4–1.6 µg/L; 1.6–1.8 µg/L; ≥1.8 µg/L); and lead (0–10 µg/L;10–20 ug/L; 20–30 ug/L; 30–40 ug/L; 40–50 ug/L; 50–60 ug/L; 60–70 ug/L; 70–80 ug/L; 80–90 ug/L; ≥90 ug/L). For each group, we graphed the mean ± SEM of eGFR, using a LOWESS (locally weighted scatterplot smoothing) curve to show the trend. To further assess the association between eGFR and metals, we divided the study cohort into different subgroups: by sex and age. Using multivariate linear regression, we analyzed the association considering the confounding factors: age, sex, CRP, and glucose (we chose to include factors that affect renal function most in the normal population. The IDF Diabetes Atlas 2020 estimates the prevalence of diabetes in the German population to be at 15.3%, which is why we included blood glucose in the multivariate regression analysis).

To study the interaction of the three metals concentrations on eGFR each metal concentrations were divided in a low and an elevated group by the median of the given metal concentration. Next, we created groups of low metal A and low metal B, low metal A and elevated metal B, elevated metal A and low metal B and finally elevated metal A and elevated metal B, see also Figs. 5, 6 and 7. Metal A and metal B stands for the individual metal whose interaction was studied. For multivariant analysis, we created groups as follows:

1. Patients having low metal A and low metal B concentrations.
2. Patients having low metal A and elevated metal B or elevated metal A and low metal B concentrations.
3. Patients having elevated metal A and elevated metal B concentrations.
4. These groups were used to test the interaction of the metal on eGFR considering confounding factors such as age, sex and fasting glucose, see Tables 6, 7 and 8.

## Electronic supplementary material

Below is the link to the electronic supplementary material.

Supplementary Material 1

## Data Availability

The original contributions presented in the study are included in the article/Supplementary Material. Further inquiries can be directed to the corresponding authors.
