# Supplementary material for: Association of whole blood heavy metal concentrations with kidney function
Source: Sci Rep. 2025 Mar 11;15:8370. doi: 10.1038/s41598-025-93548-7 (PMC11897145; doi:10.1038/s41598-025-93548-7)
Supplement: Supplementary file 1 — Supplementary Material 1 [file 41598_2025_93548_MOESM1_ESM.docx]

**Supplementary Figure**

Supplementary Figure 1. Correlation between Metals and eGFR.

**
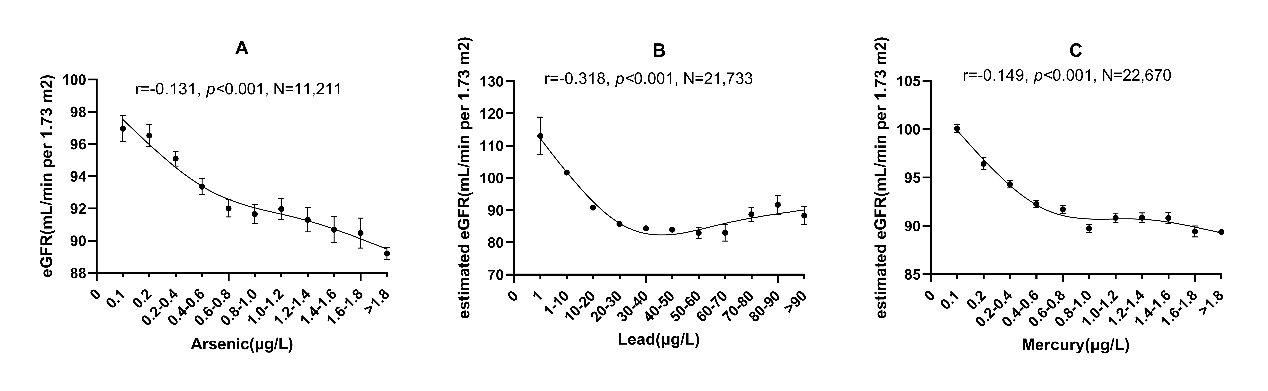
**

Metal concentrations were grouped into 11 different metal concentration groups: Arsenic and Mercury: 0.1;0.2; 0.2-0.4; 0.4-0.6; 0.6-0.8; 0.8-1.0; 1.0-1.2; 1.2-1.4; 1.4-1.6; 1.6-1.8; ≥1.8 µg/L; Lead: 1;1-10;10-20; 20-30; 30-40; 40-50; 50-60; 60-70; 70-80; 80-90; ≥90 ug/L; Each data points represent mean +/- SEM.

The ICP-MS system’s detection limits were sufficiently low to detect traces of arsenic, lead, and mercury in full blood samples. The lower limits of quantification (LLOQ) for our assays were: Arsenic (0.2 µg/L); Lead (2 µg/L); Mercury (0.2 µg/L); if the ICP-MS analysis resulted in a concentration below LLOQ, a value equal to the midpoint between zero and the LLOQ for metal samples was calculated and used in the database to avoid any bias in statistical analysis. These values were as follows: Arsenic (0.1 µg/L); Lead (1 µg/L); Mercury (0.1 µg/L).

Supplementary Figure 2. Correlation Between Metals and eGFR in Sex Subgroups.


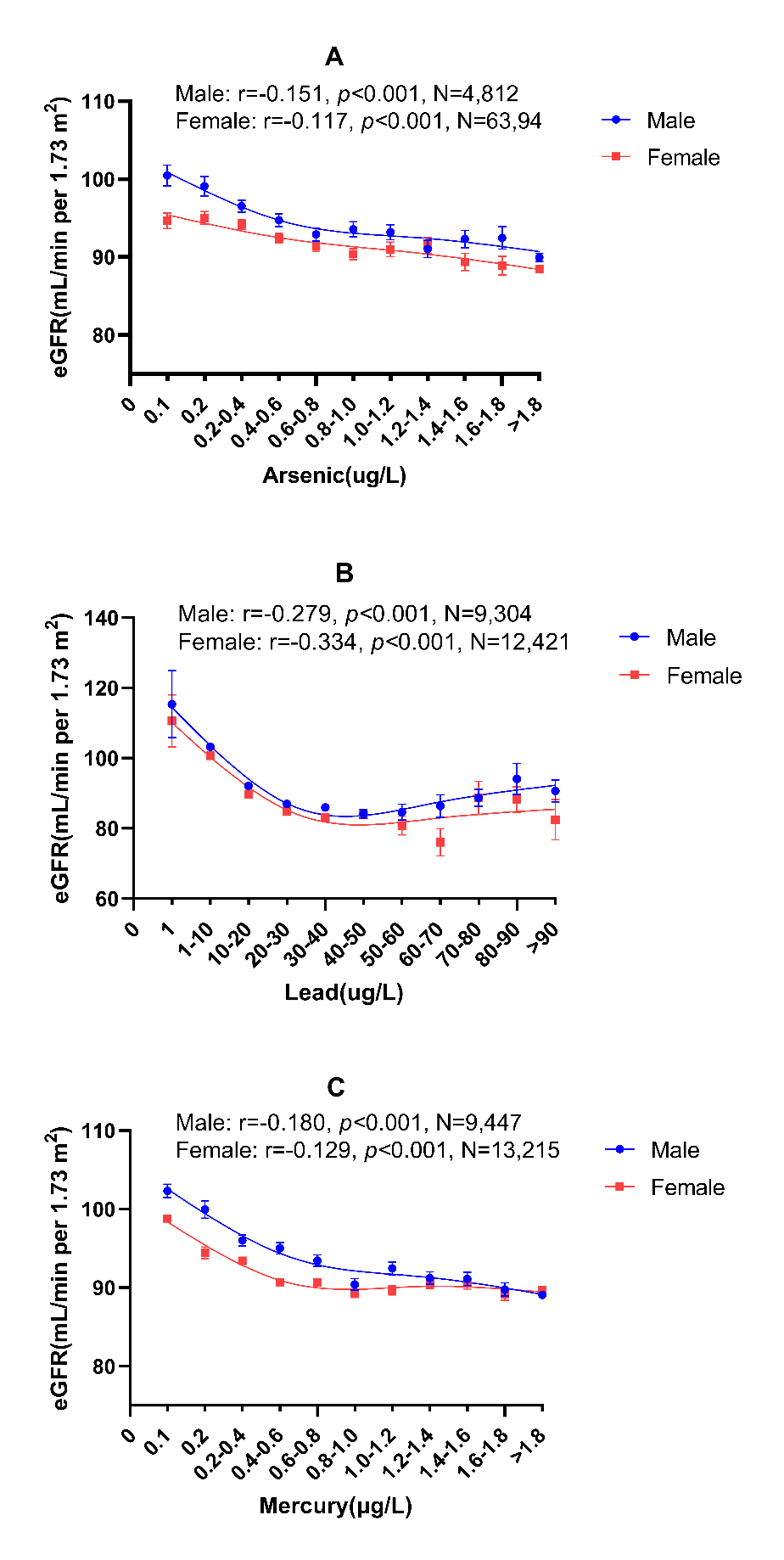


Metals were divided into eleven concentration groups (Arsenic and Mercury: 0.1;0.2; 0.2-0.4; 0.4-0.6; 0.6-0.8; 0.8-1.0; 1.0-1.2; 1.2-1.4; 1.4-1.6; 1.6-1.8; ≥1.8 µg/L); and lead (1;1-10; 10-20; 20-30; 30-40; 40-50; 50-60; 60-70; 70-80; 80-90; ≥90 ug/L). Each data points represent mean +/- SEM.

The ICP-MS system’s detection limits were sufficiently low to detect traces of arsenic, lead, and mercury in full blood samples. The lower limits of quantification (LLOQ) for our assays were: Arsenic (0.2 µg/L); Lead (2 µg/L); Mercury (0.2 µg/L); if the ICP-MS analysis resulted in a concentration below LLOQ, a value equal to the midpoint between zero and the LLOQ for metal samples was calculated and used in the database to avoid any bias in statistical analysis. These values were as follows: Arsenic (0.1 µg/L); Lead (1 µg/L); Mercury (0.1 µg/L).

Supplementary Figure 3. 3D Plot-Effects of Arsenic and Lead on eGFR in Female group**
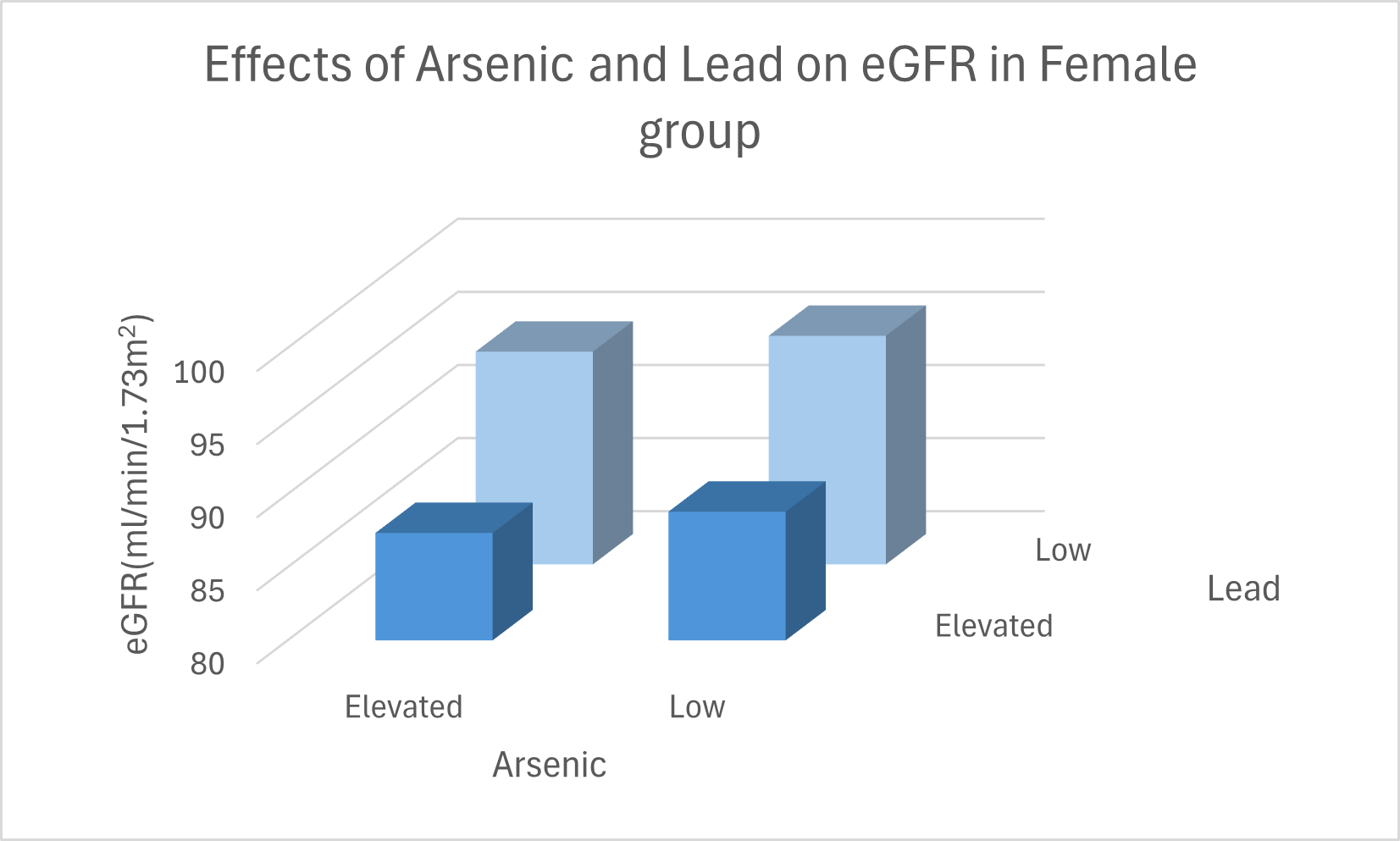
**

The patients were divided into four groups according to the median of the metal concentrations:

Group 1: Low Lead/Low Arsenic: mean±SEM: 95.63±0.194 ml/min/1.73m^2^, N=9,516

Group 2: Low Lead/ Elevated Arsenic: mean±SEM: 94.55±0.199 ml/min/1.73m2, N=8,958

Group 3: Elevated Lead/Low Arsenic: mean±SEM: 88.80±0.178 ml/min/1.73m2, N=9,857

Group 4: Elevated Lead/ Elevated Arsenic: mean±SEM: 87.35±0.176 ml/min/1.73m^2^, N=9,299

Group 1 versus group 4: *p*<0.001

Group 2 versus group 4: *p*<0.001

Group 3 versus group 4: *p*<0.001

Supplementary Figure 4. 3D Plot-Effects of Arsenic and Mercury on eGFR in Female group
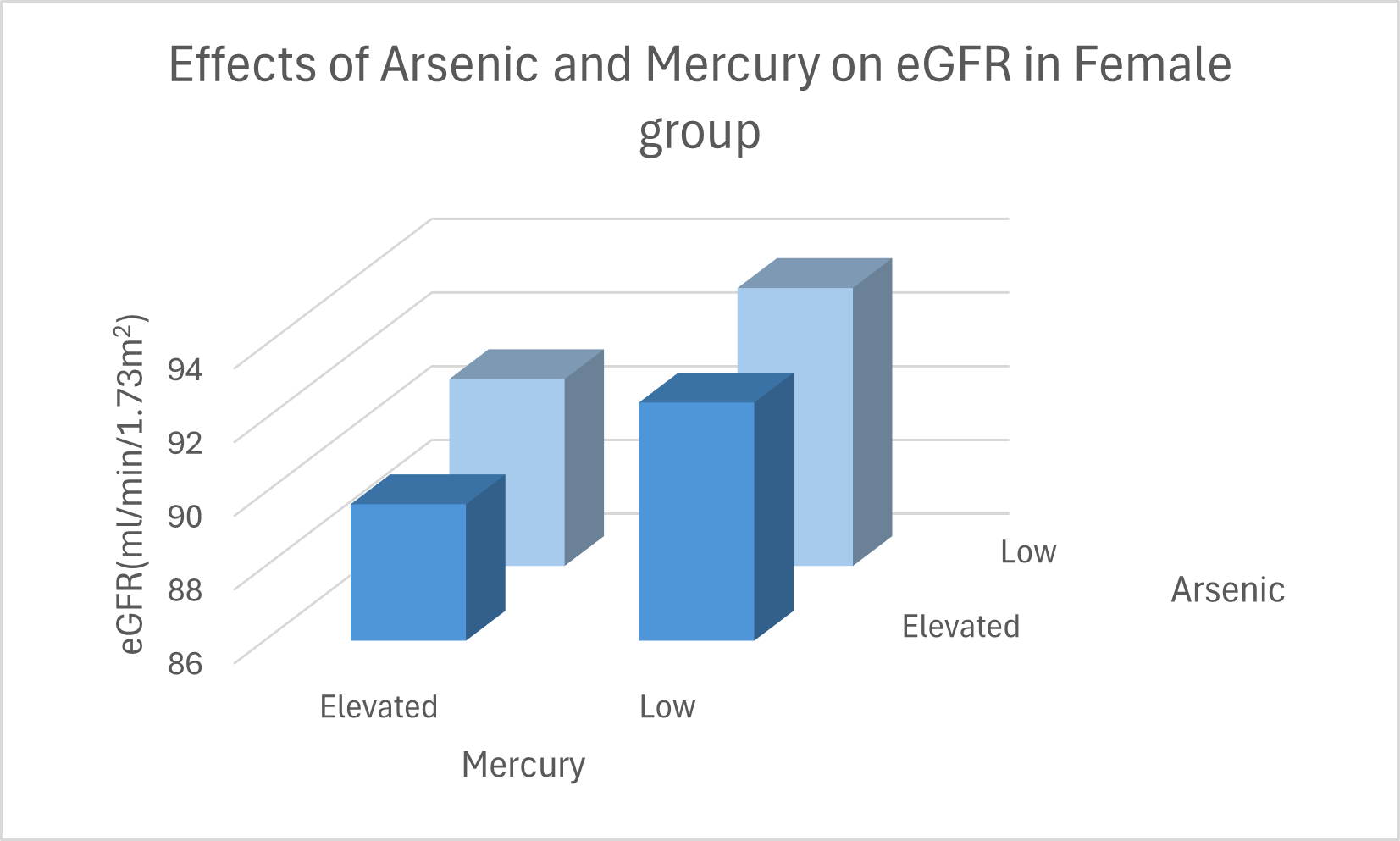


The patients were divided into four groups according to the median of the metal concentrations:

Group 1: Low Arsenic:/Low Mercury: mean±SEM: 95.54±0.187 ml/min/1.73m^2^, N=10,644

Group 2: Low Arsenic:/ Elevated Mercury: mean±SEM: 91.07±0.179 ml/min/1.73m2, N=9,523

Group 3: Elevated Arsenic:/Low Mercury: mean±SEM: 92.46±0.191 ml/min/1.73m2, N=10,086

Group 4: Elevated Arsenic:/ Elevated Mercury: mean±SEM: 89.70±0.180 ml/min/1.73m^2^, N=8,965

Group 1 versus group 4: *p*<0.001

Group 2 versus group 4: *p*<0.001

Group 3 versus group 4: *p*<0.001

Supplementary Figure 5. 3D Plot-Effects of Lead and Mercury on eGFR in Female group
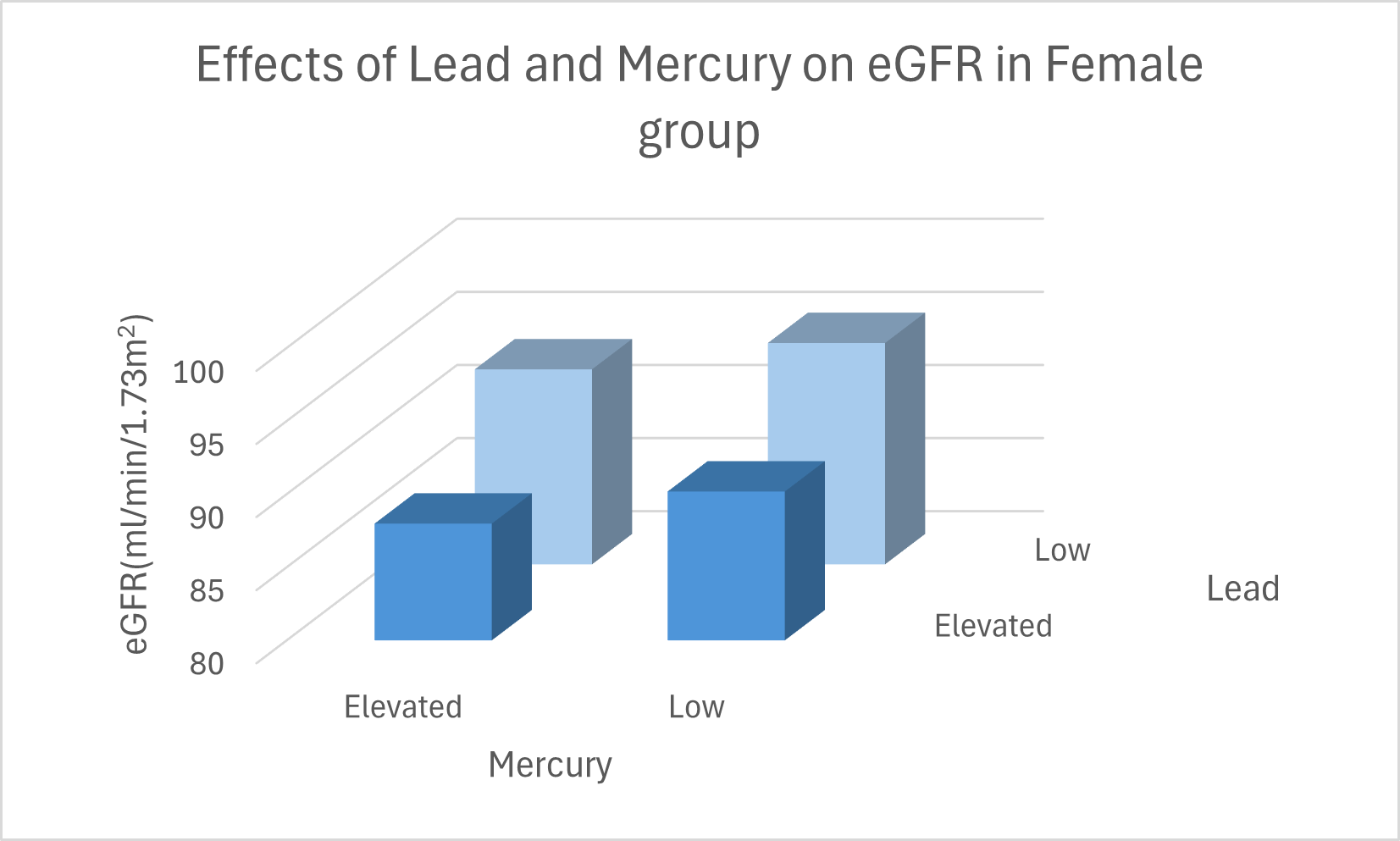


The patients were divided into four groups according to the median of the metal concentrations:

Group 1: Low Mercury /Low Lead: mean±SEM: 95.14±0.169 ml/min/1.73m^2^, N=13,208

Group 2: Low Mercury / Elevated Lead: mean±SEM: 90.18±0.160 ml/min/1.73m2, N=13,549

Group 3: Elevated Mercury /Low Lead: mean±SEM: 93.34±0.166 ml/min/1.73m2, N=12,087

Group 4: Elevated Mercury / Elevated Lead: mean±SEM: 87.98±0.151 ml/min/1.73m^2^, N=12,428

Group 1 versus group 4: *p*<0.001

Group 2 versus group 4: *p*<0.001

Group 3 versus group 4: *p*<0.001

Supplementary Figure 6. 3D Plot-Effects of Arsenic and Lead on eGFR in Male group


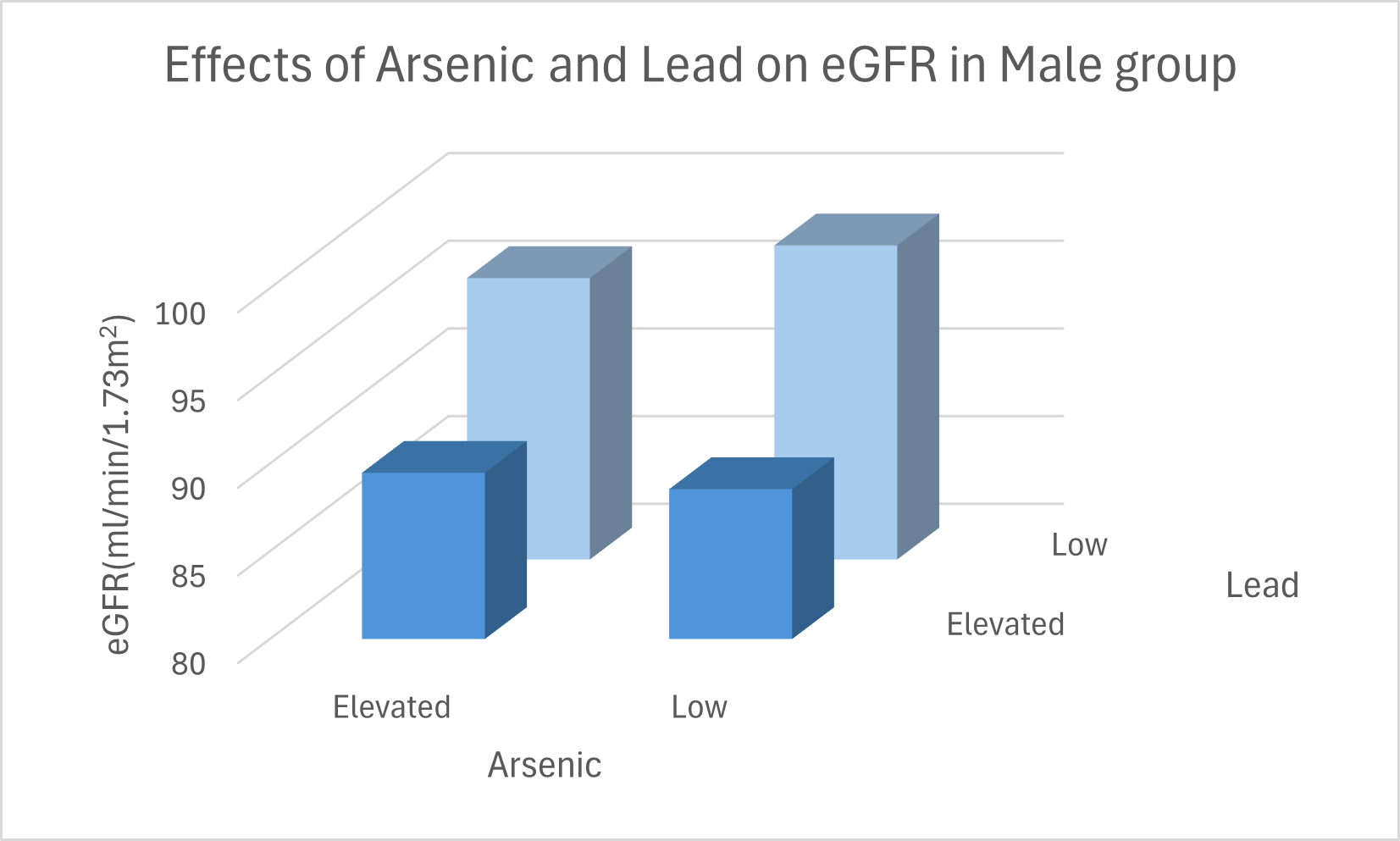


The patients were divided into four groups according to the median of the metal concentrations:

Group 1: Low Lead/Low Arsenic: mean±SEM: 97.91±0.267 ml/min/1.73m^2^, N=6,357

Group 2: Low Lead/ Elevated Arsenic: mean±SEM: 96.05±0.258 ml/min/1.73m2, N=6,589

Group 3: Elevated Lead/Low Arsenic: mean±SEM: 88.55±0.252 ml/min/1.73m2, N=5,239

Group 4: Elevated Lead/ Elevated Arsenic: mean±SEM: 89.485±0.207 ml/min/1.73m^2^, N=7,761

Group 1 versus group 4: *p*<0.001

Group 2 versus group 4: *p*<0.001

Group 3 versus group 4: *p*<0.001

Supplementary Figure 7. 3D Plot-Effects of Arsenic and Mercury on eGFR in Male group


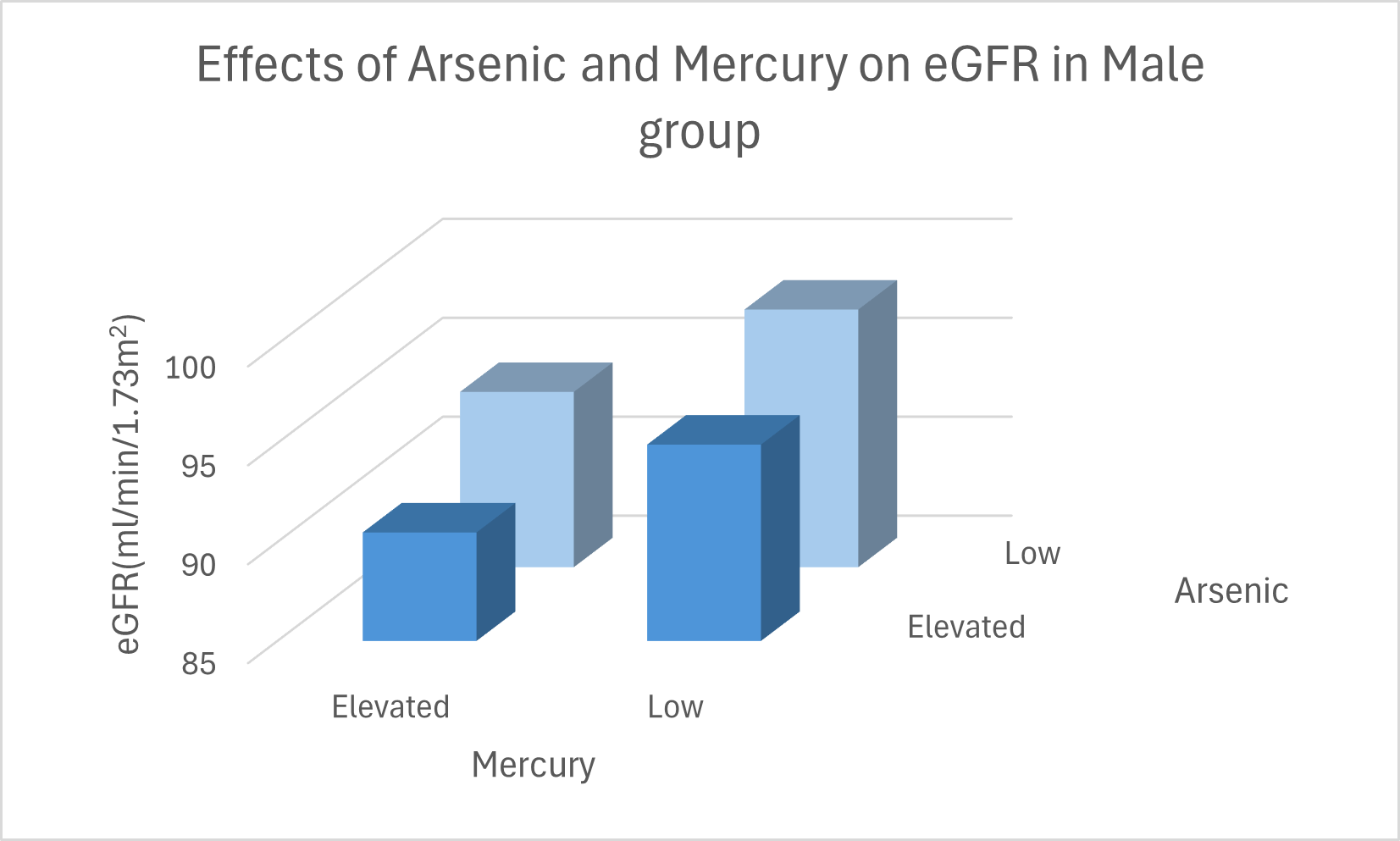


The patients were divided into four groups according to the median of the metal concentrations:

Group 1: Low Arsenic /Low Mercury: mean±SEM: 98.02±0.250 ml/min/1.73m^2^, N=8,109

Group 2: Low Arsenic / Elevated Mercury: mean±SEM: 93.86±0.207 ml/min/1.73m2, N=9,472

Group 3: Elevated Arsenic /Low Mercury: mean±SEM: 94.93±0.264 ml/min/1.73m2, N=6,565

Group 4: Elevated Arsenic / Elevated Mercury: mean±SEM: 90.49±0.200 ml/min/1.73m^2^, N=7,928

Group 1 versus group 4: *p*<0.001

Group 2 versus group 4: *p*<0.001

Group 3 versus group 4: *p*<0.001

Supplementary Figure 8. 3D Plot-Effects of Lead and Mercury on eGFR in Male group


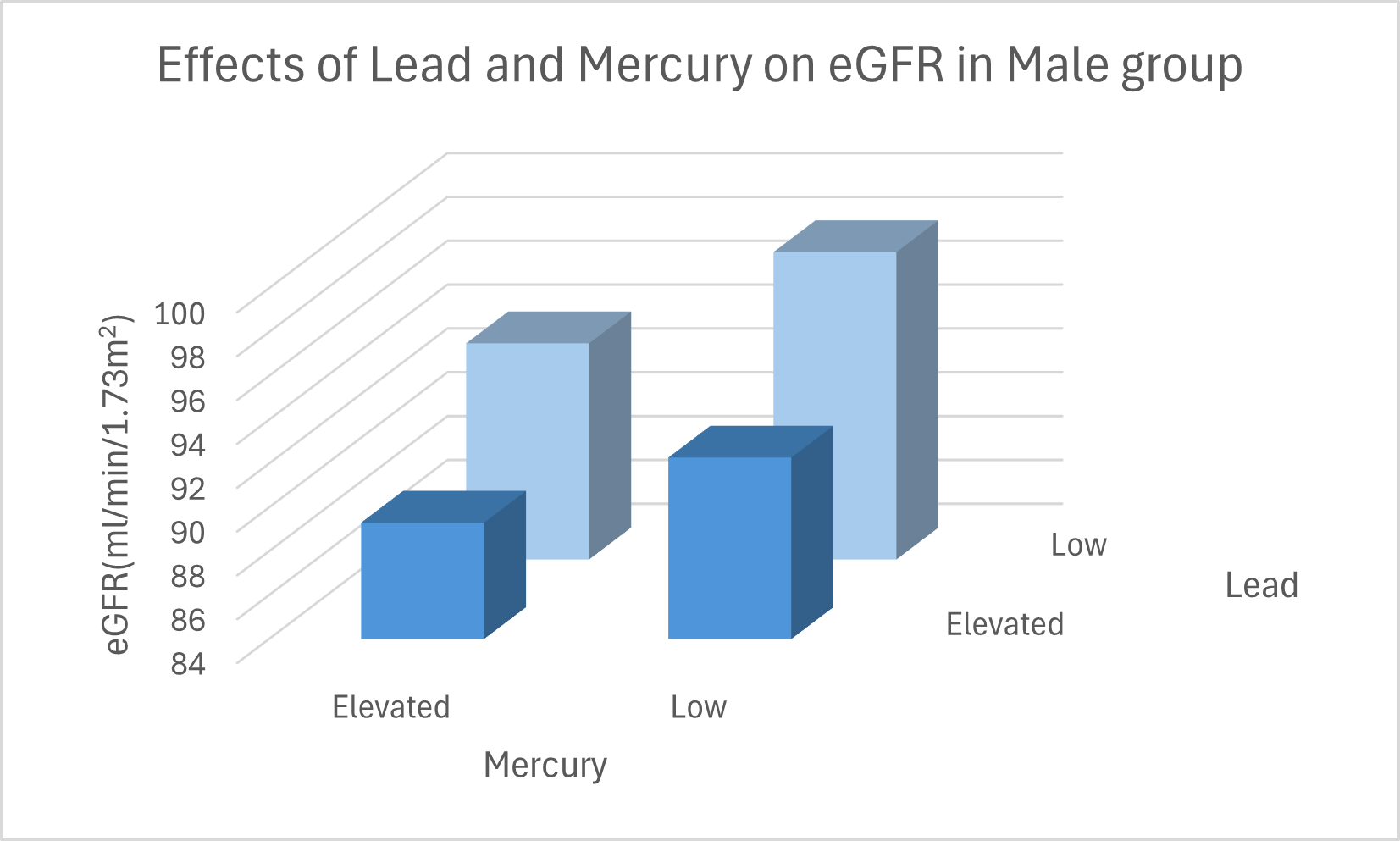


The patients were divided into four groups according to the median of the metal concentrations:

Group 1: Low Mercury Low Lead: mean±SEM: 98.03±0.250 ml/min/1.73m^2^, N=8,109

Group 2: Low Mercury / Elevated Lead: mean±SEM: 92.28±0.216 ml/min/1.73m2, N=9,281

Group 3: Elevated Mercury /Low Lead: mean±SEM: 93.86±0.207 ml/min/1.73m2, N=9,472

Group 4: Elevated Mercury / Elevated Lead: mean±SEM: 89.31±0.174 ml/min/1.73m^2^, N=10,644

Group 1 versus group 4: *p*<0.001

Group 2 versus group 4: *p*<0.001

Group 3 versus group 4: *p*<0.001

Supplementary Figure 9. 3D Plot-Effects of Arsenic and Lead on eGFR in Elderly group (age above median of the study population)**
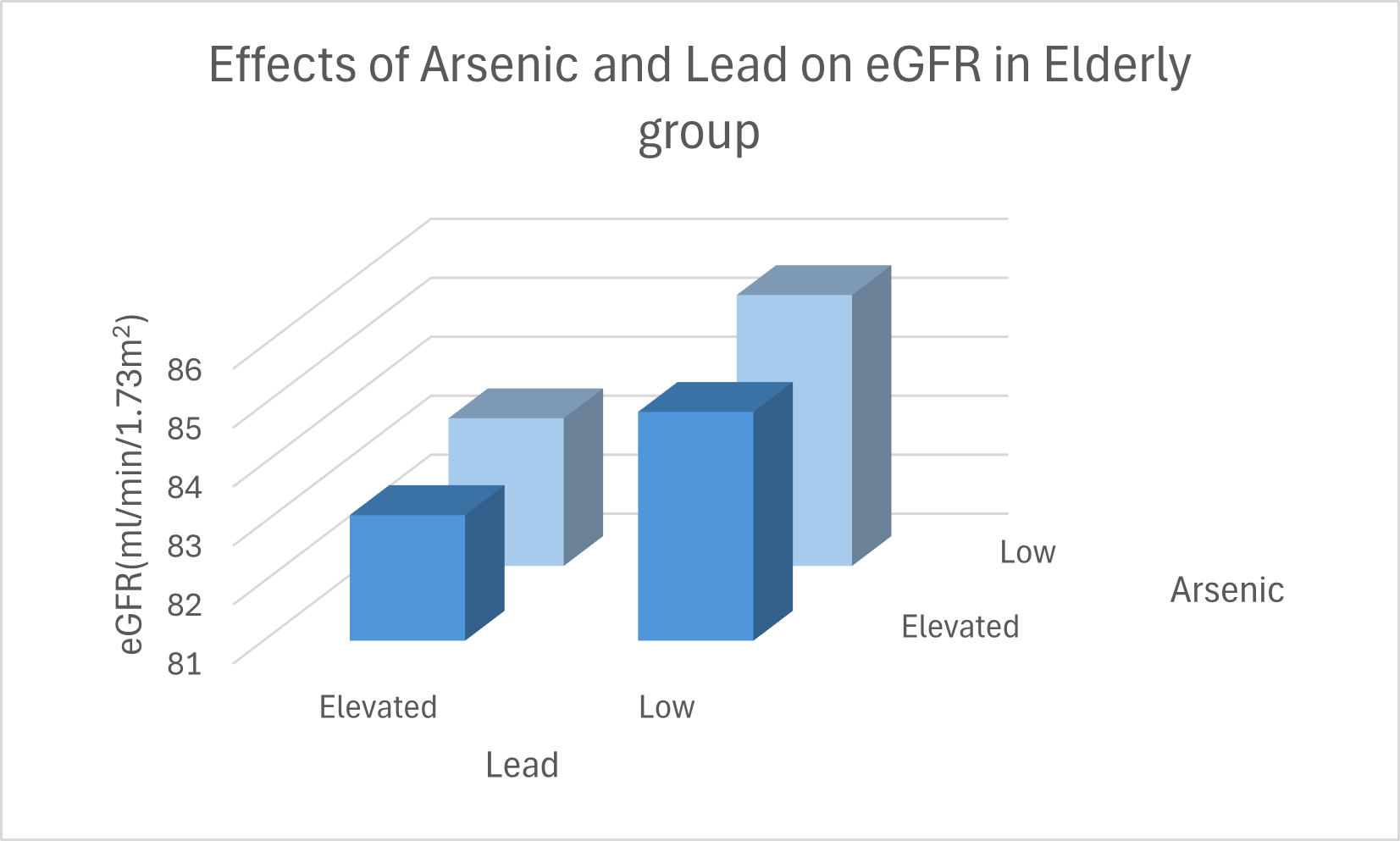
**

The patients were divided into four groups according to the median of the metal concentrations:

Group 1: Low Lead/Low Arsenic: mean±SEM: 85.59±0.178 ml/min/1.73m^2^, N=6,682

Group 2: Low Lead/ Elevated Arsenic: mean±SEM: 84.88±0.174 ml/min/1.73m2, N=7,045

Group 3: Elevated Lead/Low Arsenic: mean±SEM: 83.50±0.142 ml/min/1.73m2, N=11,042

Group 4: Elevated Lead/ Elevated Arsenic: mean±SEM: 83.13±0.140ml/min/1.73m^2^, N=11,045

Group 1 versus group 4: *p*<0.001

Group 2 versus group 4: *p*<0.001

Group 3 versus group 4: *p*<0.001

Supplementary Figure 10. 3D Plot-Effects of Arsenic and Mercury on eGFR in Elderly group (age above median of the study population)
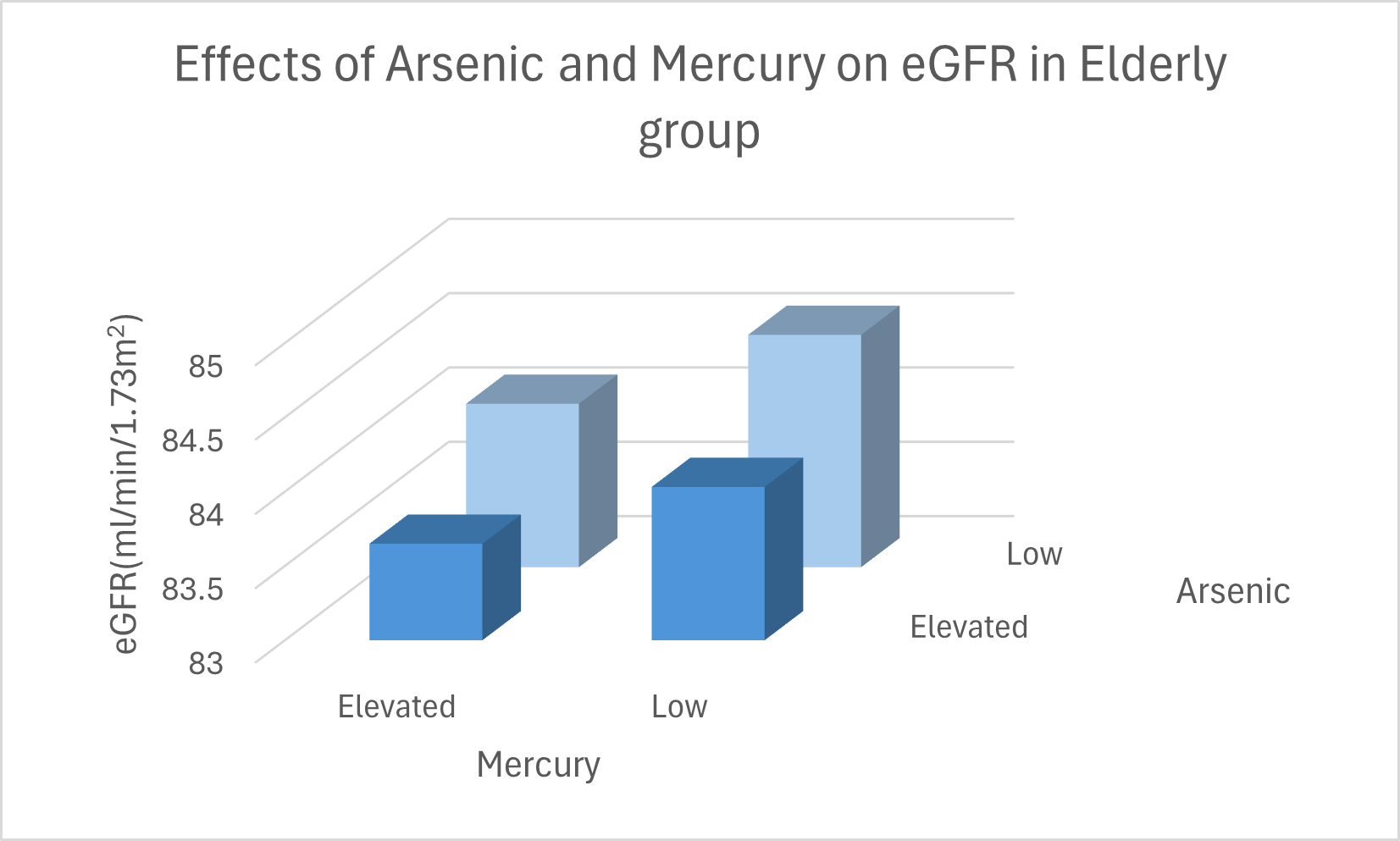


The patients were divided into four groups according to the median of the metal concentrations:

Group 1: Low Arsenic/Low Mercury: mean±SEM: 84.56±0.166 ml/min/1.73m^2^, N=8,391

Group 2: Low Arsenic / Elevated Mercury: mean±SEM: 84.10±0.147 ml/min/1.73m2, N=9,615

Group 3: Elevated Arsenic /Low Mercury: mean±SEM: 84.03±0.162 ml/min/1.73m2, N=8,754

Group 4: Elevated Arsenic / Elevated Mercury: mean±SEM: 83.65±0.144 ml/min/1.73m^2^, N=9,978

Group 1 versus group 4: *p*<0.001

Group 2 versus group 4: *p*<0.001

Group 3 versus group 4: *p*<0.001

Supplementary Figure 11. 3D Plot-Effects of Lead and Mercury on eGFR in Elderly group (age above median of the study population)


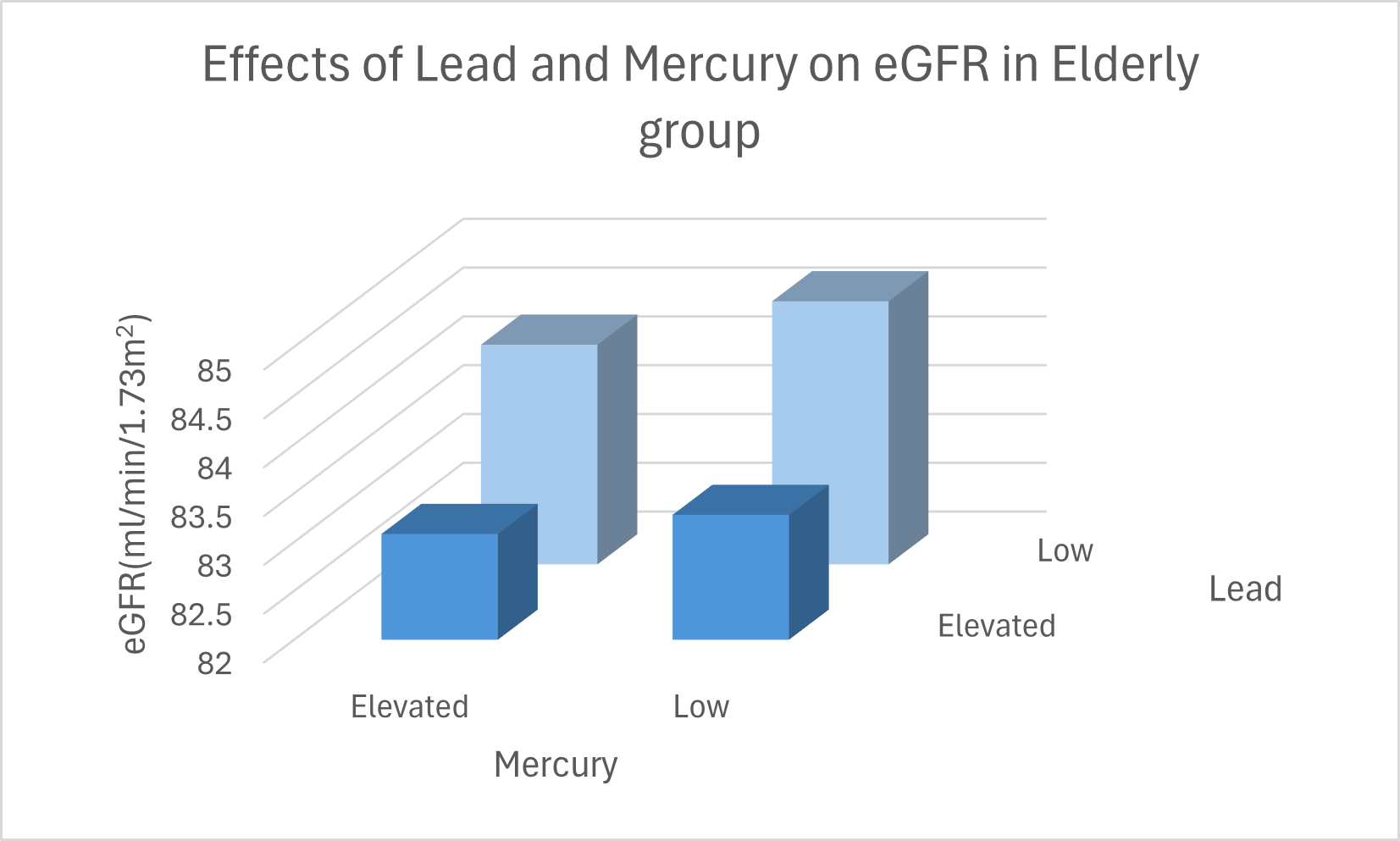


The patients were divided into four groups according to the median of the metal concentrations:

Group 1: Low Mercury/Low Lead: mean±SEM: 84.69±0.159 ml/min/1.73m^2^, N=9,168

Group 2: Low Mercury / Elevated Lead: mean±SEM: 83.28±0.132 ml/min/1.73m2, N=13,528

Group 3: Elevated Mercury/ Low Lead: mean±SEM: 84.25±0.141 ml/min/1.73m2, N=10,392

Group 4: Elevated Mercury / Elevated Lead: mean±SEM: 83.08±0.121 ml/min/1.73m^2^, N=14,752

Group 1 versus group 4: *p*<0.001

Group 2 versus group 4: *p*<0.001

Group 3 versus group 4: *p*<0.001

Supplementary Figure 12. 3D Plot-Effects of Arsenic and Lead on eGFR in Younger group (age below median of the study population)


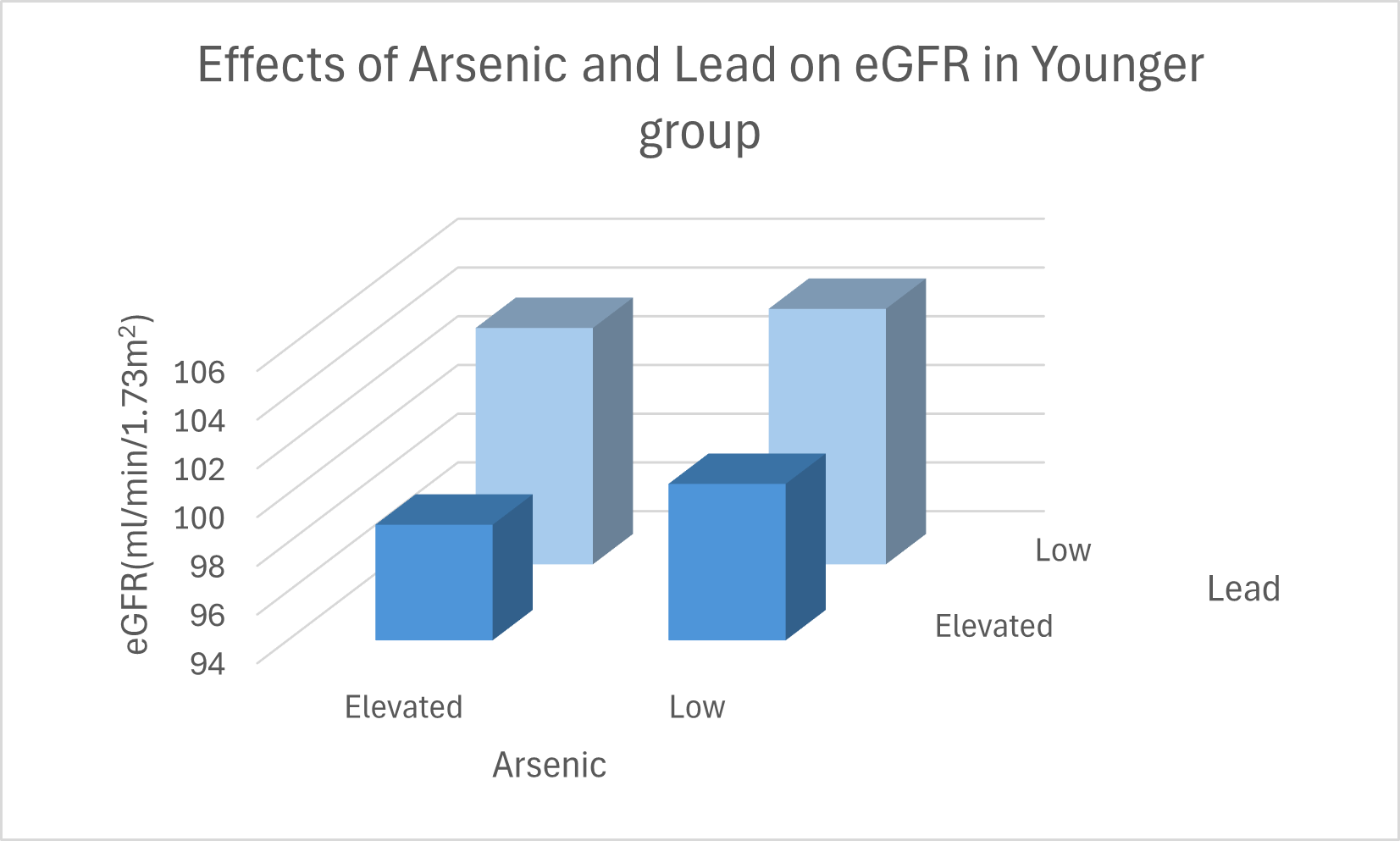


The patients were divided into four groups according to the median of the metal concentrations and age:

Group 1: Low Lead/Low Arsenic: mean±SEM: 104.49±0.205 ml/min/1.73m^2^, N=9,198

Group 2: Low Lead/ Elevated Arsenic: mean±SEM: 103.70±0.211 ml/min/1.73m2, N=8,508

Group 3: Elevated Lead/Low Arsenic: mean±SEM: 100.42±0.235 ml/min/1.73m2, N=6,353

Group 4: Elevated Lead/ Elevated Arsenic: mean±SEM: 98.75±0.239 ml/min/1.73m^2^, N=5,663

Group 1 versus group 4: *p*<0.001

Group 2 versus group 4: *p*<0.001

Group 3 versus group 4: *p*<0.001

Supplementary Figure 13. 3D Plot-Effects of Arsenic and Mercury on eGFR in Younger group (age below median of the study population)


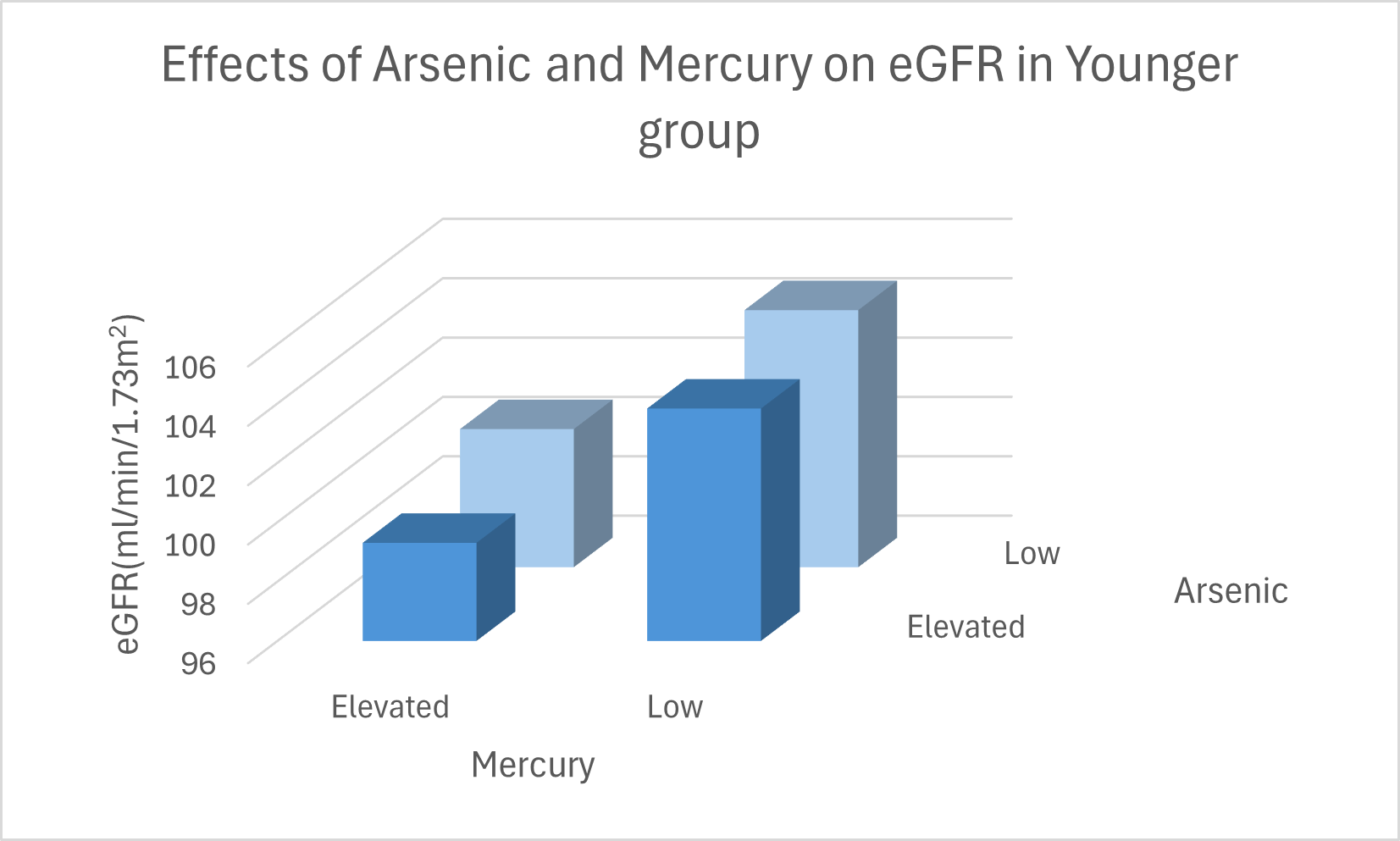


The patients were divided into four groups according to the median of the metal concentrations and age:

Group 1: Low Arsenic/Low Mercury: mean±SEM: 104.66±0.215 ml/min/1.73m^2^, N=8,594

Group 2: Low Arsenic / Elevated Mercury: mean±SEM: 100.65±0.206 ml/min/1.73m2, N=7,612

Group 3: Elevated Arsenic /Low Mercury: mean±SEM: 103.83±0.222 ml/min/1.73m2, N=7,904

Group 4: Elevated Arsenic / Elevated Mercury: mean±SEM: 99.31±0.208 ml/min/1.73m^2^, N=6,922

Group 1 versus group 4: *p*<0.001

Group 2 versus group 4: *p*<0.001

Group 3 versus group 4: *p*<0.001

Supplementary Figure 14. 3D Plot-Effects of Lead and Mercury on eGFR in Younger group (age below median of the study population)


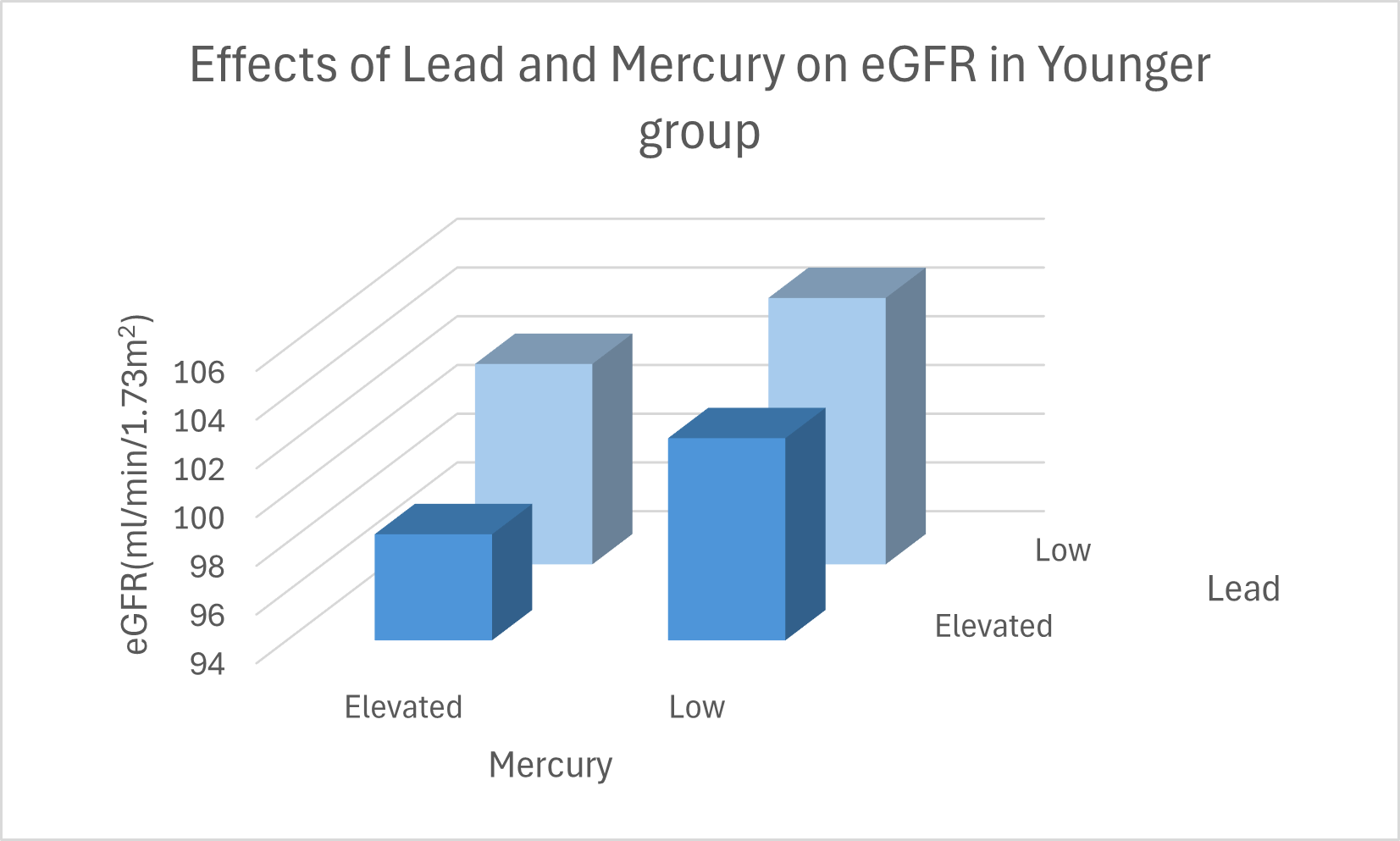


The patients were divided into four groups according to the median of the metal concentrations and age:

Group 1: Low Mercury/Low Lead: mean±SEM: 104.93±0.182 ml/min/1.73m^2^, N=12,157

Group 2: Low Mercury / Elevated Lead: mean±SEM: 102.30±0.204 ml/min/1.73m2, N=9,312

Group 3: Elevated Mercury /Low Lead: mean±SEM: 102.23±0.179 ml/min/1.73m2, N=11,175

Group 4: Elevated Mercury / Elevated Lead: mean±SEM: 98.35±0.190 ml/min/1.73m^2^, N=8,330

Group 1 versus group 4: *p*<0.001

Group 2 versus group 4: *p*<0.001

Group 3 versus group 4: *p*<0.001

**Supplementary Tables**

Supplementary Table 1. Multivariate Linear Regression – Arsenic (Dependent Variable: eGFR) in eGFR<60 (mL/min per 1.73 m^2^) group

| **Variables** | **Multivariate Linear Regression** | |
| --- | --- | --- |
|  | **β Coefficient / 95%CI** | ***p* Value** |
| Constant | 47.713 (32.996~62.430) | <0.001 |
| Age | -0.019 (-0.174~-0.137) | 0.814 |
| Sex | 0.702 (-3.480~-4.883) | 0.740 |
| CRP | -0.2733 (-0.523~-0.024) | 0.032 |
| Glucose | 0.029 (-0/033~0.091) | 0.354 |
| Arsenic | 0.195 (-0.509~0.900) | 0.583 |

Supplementary Table 2. Multivariate Linear Regression – Arsenic (Dependent Variable: eGFR) in eGFR≥60 (mL/min per 1.73 m^2^) group

| **Variables** | **Multivariate Linear Regression** | |
| --- | --- | --- |
|  | **β Coefficient / 95%CI** | ***p* Value** |
| Constant | 139.766 (136.634~142.898) | <0.001 |
| Age | -0.720 (-0.747~-0.693) | <0.001 |
| Sex | -2.102 (-2.980~-1.224) | <0.001 |
| CRP | -0.013 (-0.062~0.036) | 0.606 |
| Glucose | -0.055 (-0.076~-0.033) | <0.001 |
| Arsenic | -0.213 (-0.418~-0.008) | 0.042 |

Supplementary Table 3. Multivariate Linear Regression – Lead (Dependent Variable: eGFR) in eGFR<60 (mL/min per 1.73 m^2^) group

| **Variables** | **Multivariate Linear Regression** | |
| --- | --- | --- |
|  | **β Coefficient / 95%CI** | ***p* Value** |
| Constant | 51.490 (42.413~60.567) | <0.001 |
| Age | -0.037 (-0.133~0.058) | 0.441 |
| Sex | 0.985 (-1.525~3.496) | 0.440 |
| CRP | -0.060 (-0.156~0.036) | 0.219 |
| Glucose | -0.011 (-0.049~0.028) | 0.588 |
| Lead | 0.037 (-0.066~0.139) | 0.483 |

Supplementary Table 4. Multivariate Linear Regression – Lead (Dependent Variable: eGFR) in eGFR≥60 (mL/min per 1.73 m^2^) group

| **Variables** | **Multivariate Linear Regression** | |
| --- | --- | --- |
|  | **β Coefficient / 95%CI** | ***p* Value** |
| Constant | 137.522 (135.291~139.754) | <0.001 |
| Age | -0.734(-0.754~-0.714) | <0.001 |
| Sex | -1.633 (-2.268~-0.998) | <0.001 |
| CRP | -0.029 (-0.063~0.005) | 0.096 |
| Glucose | -0.035 (-0.050~-0.020) | <0.001 |
| Lead | -0.025 (-0.051~-0.653×10-^4^) | 0.051 |

Supplementary Table 5. Multivariate Linear Regression – Mercury (Dependent Variable: eGFR) in eGFR<60 (mL/min per 1.73 m^2^) group

| **Variables** | **Multivariate Linear Regression** | |
| --- | --- | --- |
|  | **β Coefficient / 95%CI** | ***p* Value** |
| Constant | 54.195 (44.774~63.616) | <0.001 |
| Age | -0.054 (-0.154~0.046) | 0.291 |
| Sex | 0.721 (-1.870~3.311) | 0.584 |
| CRP | -0.060 (-0.158~0.037) | 0.224 |
| Glucose | -0.015 (-0.055~0.025) | 0.454 |
| Mercury | -0.062 (-0.533~0.409) | 0.795 |

Supplementary Table 6. Multivariate Linear Regression – Mercury (Dependent Variable: eGFR) in eGFR≥60 (mL/min per 1.73 m^2^) group

| **Variables** | **Multivariate Linear Regression** | |
| --- | --- | --- |
|  | **β Coefficient / 95%CI** | ***p* Value** |
| Constant | 137.974 (135.773~140.174) | <0.001 |
| Age | -0.735 (-0.753~-0.716) | <0.001 |
| Sex | -1.860 (-2.482~-1.239) | <0.001 |
| CRP | -0.026 (-0.060~0.009) | 0.144 |
| Glucose | -0.035 (-0.050~-0.020) | <0.001 |
| Mercury | -0.337 (-0.463~0.211) | <0.001 |

Supplementary Table 7: Overview of Clinical Trials

| Study Title | Study Design | Cohort Size and Place | Kidney Function Biomarker | Key Findings |
| --- | --- | --- | --- | --- |
| Sex-Specific Associations of Urinary Metals with Renal Function: A Cross-sectional Study in China. 2023 May. DOI: 10.1007/s12011-022-03349-6 (1) | Cross-sectional Study | 2,775 participants above the age of 30; no cancer or tumor; eGFR≥60 mL/min/1.73 m^2^ in Guangxi China | eGFR | Urinary arsenic, cadmium, lead, manganese, and chromium were together positively associated with eGFR. Lead showed the strongest effects, especially in women. |
| Association of Blood Heavy Metal Levels and Renal Function in Korean Adults. 2022 May. DOI: 10.3390/ijerph19116646 (2) | Cross-sectional Study | 1984 participants above the age of 19; | eGFR | Analysis of the correlation between blood lead, mercury, cadmium, and nickel levels and blood eGFR levels revealed that blood eGFR levels were significantly negatively correlated with blood lead, cadmium, and nickel levels. |
| Association and mediation analyses among multiple metals exposure, plasma folate, and community-based impaired estimated glomerular filtration rate in central Taiwan. 2022 Apr. DOI: 10.1186/s12940-022-00855-x (3) | Cross-sectional study | 1,643 participants who lived locally for >5 years and were >40 years old in Taiwan | eGFR | Arsenic, lead, cadmium, chromium, and nickel together were associated with an impaired eGFR – of these five especially arsenic, lead, and cadmium. |
| Associations among Heavy Metals and Proteinuria and Chronic  Kidney Disease. 2021. Jan.  DOI: 10.3390/diagnostics11020282 (4) | Observational study | 2,447 participants in Southern Taiwan | eGFR  Proteinuria was measured using reagent strips | High blood lead and high urine nickel, manganese, copper, and cadmium were significantly associated with proteinuria. Interactions between blood lead and urine Cr, and between urine lead and copper affected proteinuria significantly. Participants with high blood lead and high urine copper were significantly associated with an eGFR of <60 mL/min/1.73 m2. |
| Prospective associations between environmental heavy metal exposure and renal outcomes in adults with chronic kidney disease. 2018 Sep. DOI: 10.1111/nep.13089 (5) | Prospective observational study | 2,343 CKD patients (533 with progression to end-stage renal disease [ESRD] and 1810 without) in Taiwan |  | Patients with CKD and long-term exposure to soil-based heavy metals (after consideration of confounders only zinc and nickel), had rapid progression to ESRD. |
| Arsenic Exposure from Drinking Water and the Incidence of CKD in Low to Moderate Exposed Areas of Taiwan: A 14-Year Prospective Study. 2017 Dec.  DOI: 10.1053/j.ajkd.2017.06.012 (6) | Prospective observational study | 6,093 participants ≥40 years in Taiwan | Proteinuria | Higher creatinine-adjusted urinary total concentrations of arsenic were associated with a higher prevalence of proteinuria. |
| End-stage renal disease after occupational lead exposure: 20 years of follow-up. 2017 Jun. DOI: 10.1136/oemed-2016-103876 (7) | Comparative Study | 10,303 lead-workers in Sweden |  | 30 (0.29%) individuals of the cohort developed ESRD during the median follow-up period of 26.3 years. This study shows no statistically significant association between lead exposure (following the current occupational recommendations for Sweden) and ESRD. |
| Urinary KIM-1: a novel biomarker for evaluation of occupational exposure to lead. (8)  2016 Dec.  DOI:10.1038/srep38930 | Retrospective study | 184 participants  (92 subjects with occupational lead exposure; 92 healthy adults with no history of lead exposure as controls) in China | Albumin; KIM-1(urinary kidney injury molecule-1); *α*1-MG (*α*1-microglobulin); *β*2-MG (*β*2-microglobulin); NAG (N-acetyl-*β*-(D)-glucosaminidase) | Short-period occupational lead exposure may cause injury of renal tubules. Urinary KIM-1 demonstrated a better relationship with blood lead levels than other traditional urinary biomarkers of kidney injury, including NAG, α1-MG, β2-MG. |
| The association of urine arsenic with prevalent and incident chronic kidney disease: evidence from the Strong Heart Study. 2015 Jul. (9) DOI: 10.1097/EDE.0000000000000313 (9) | Cross-sectional study | 3,851 American Indians aged 45-74 years | eGFR | The inverse association of inorganic arsenic in urine with prevalent CKD suggests that kidney disease affects excretion of inorganic arsenic. Arsenic species were positively associated with incident CKD. |
| Environmental Heavy Metal Exposure and Chronic Kidney  Disease in the General Population 2015. May.  DOI: 10.3346/ jkms.2015.30.3.272 (10) | Cross-sectional study | 1,797 participants (of these, 128 (7.1%) had CKD) in Korea | eGFR; ACR (albumin creatinine ratio) | Environmental low level of lead, mercury, and cadmium exposure in the general population was not associated with CKD. However, cadmium exposure was associated with CKD, especially in adults with hypertension or diabetes. |
| End-stage renal disease and low-level exposure to lead, cadmium and mercury; a population-based, prospective nested case-referent study in Sweden. (11) 2013 Jan. DOI: 10.1186/1476-069X-12-9 | Prospective study | 496 participants  (118 CKD cases and 378 matched) in Sweden |  | Erythrocyte lead is associated with end-stage renal disease, but further studies are needed to evaluate causality. Gender-specific analyses suggest potential differences in susceptibility or in exposure biomarker reliability. |
| Blood Cadmium and Lead and Chronic Kidney Disease in US Adults: A Joint Analysis.(12) 2009 Nov. DOI: 10.1093/aje/kwp248 | Cross-sectional study | 14,778 adults aged ≥20 years in USA | Albuminuria and eGFR | Cadmium and lead may be considered as risk factors for chronic kidney disease in the general population. |
| Renal Effects of Dental Amalgam in Children: The New England Children’s Amalgam Trial. (13)  2008 Mar 23. DOI: 10.1289/ehp.10504  (mercury) | Randomized controlled study | 534 children aged 6-10 years with two or more posterior teeth with caries but no prior amalgam restorations in USA | Albumin, α-1-microglobulin (A1M), γ-glutamyl transpeptidase (γ-GT), and (NAG) | There were no significant differences between treatment groups regarding average levels of renal biomarkers, nor significant effects of the number of dental amalgams on these markers. |
| Low-level environmental exposure to lead and progressive chronic kidney diseases. (14)  2006 Aug. DOI: 10.1016/j.amjmed.2006.01.005 | Randomized Controlled study | 108 CKD patients with low-normal body lead burden (BLB) and no lead exposure history in Taiwan | Serum creatinine | Environmental exposure to lead, even at low level, may accelerate progressive renal insufficiency in nondiabetic patients with CKD. |
| Environmental exposure to lead and progressive diabetic nephropathy in patients with type II diabetes.(15)  2006 Jun. DOI: 10.1038/sj.ki.5001505 | Randomized Controlled study | 87 patients with type two diabetes and diabetic nephropathy, normal body lead burden and no lead exposure history in Taiwan. | Serum creatinine | Low-level environmental lead exposure accelerates progressive diabetic nephropathy. Lead-chelation therapy can decrease its rate of progression. |
| Environmental exposure to lead and progression of chronic renal diseases: a four-year prospective longitudinal study. (16) 2004 Apr. DOI: 10.1097/01.asn.0000118529.01681.4f | Prospective study | 121 adults with chronic renal insufficiency (serum creatinine 1.5 mg/dL-3.9mg/dL) in Taiwan | GFR | Each increase of 10μg in the body lead burden (BLB) or 1μg/dl in the blood lead level (BLL) reduced the GFR by 1.3 (P = 0.002) or 4.0 ml/min (P = 0.01) during the study period. Low-level environmental lead exposure is associated with an accelerated deterioration of renal insufficiency. Even at levels far below the normal ranges, both increased BLL and BLB predict an accelerated progression of chronic renal diseases. |
| Environmental lead exposure and progression of chronic renal diseases in patients without diabetes. (17)  2003 Jan. DOI: 10.1056/NEJMoa021672 | Observational study | 202 patients with chronic renal insufficiency, normal total-body lead burden and no history of exposure to lead in Taiwan | Serum creatinine | Low-level environmental lead exposure may accelerate progressive renal insufficiency in patients without diabetes who have chronic renal diseases. Repeated chelation therapy may improve renal function and slow the progression of renal insufficiency. |
| Environmental lead exposure and progressive renal insufficiency.(18)  2001 Jan. DOI: 10.1001/archinte.161.2.264 | Prospective controlled study | 110 patients with chronic renal insufficiency, normal BLB and without a history of previous heavy lead exposure in Taiwan | Serum creatinine level | Long-term low-level environmental lead exposure may subtly affect progressive renal insufficiency in the general population. Progressive renal insufficiency may be improved at least 1 year after lead chelating therapy. |
| Chelation therapy for patients with elevated body lead burden and progressive renal insufficiency. A randomized, controlled trial. (19)  1999 Jan. DOI: 10.7326/0003-4819-130-1-199901050-00003 | Randomized controlled study | 32 patients with chronic renal insufficiency, mildly elevated body lead burden via 72-hour urine collection and < 2.90 μmol of lead in 72h urine collection and no history of heavy lead exposure in Taiwan | Serum creatinine | Long-term exposure to low levels of environmental lead may be associated with impaired renal function in patients with chronic renal diseases. |
| Usefulness of determining urinary markers of early renal damage for monitoring nephrotoxicity during occupational exposure to mercury vapors.(20)  1998. PMID: 9695060 | Case-control study | 83 males with history of occupational exposure to metallic mercury vapors from 0.6 to 37 years, and 30 non-exposed males in Poland | Albumin, IgG, transferrin, retinol binding protein (RBP) alpha 1-microglobulin | The determination of proteins in urine as markers of early renal damage may be useful for monitoring occupational exposure to mercury vapors, especially in a group of workers with elevated levels of urine mercury concentrations. |
| Effects of Exposure to Elemental Mercury on the Nervous System and the Kidneys of Workers Producing Natural Gas.  1996 Mar-Apr. DOI: 10.1080/00039896.1996.9936002. (21) | Cohort study | 18 males involved regularly in cleaning activities at gas-production sites in the Netherlands | N-acetyl-beta-D-glucosaminidase | After exposure to mercury at levels below the biological exposure index, a transient increase in N-acetyl-beta-D-glucosaminidase can be observed but is not an early indicator for the development of renal dysfunction. |
| High lead content of deciduous teeth in chronic renal failure. 1991 Nov. DOI: 10.1007/BF00857878 (22) | Comparative Study | 22 children aged 5-14 years at different stages of chronic renal failure (CRF) and 36 controls in Germany |  | Mean dental Pb was significantly higher in patients than controls. No difference in blood Pb levels between cases and controls. |

1. Lin Y, Cai J, Liu Q, Mo X, Xu M, Zhang J, et al. Sex-Specific Associations of Urinary Metals with Renal Function: a Cross-sectional Study in China. Biological trace element research. 2023;201(5):2240-9.

2. Park Y, Lee SJ. Association of Blood Heavy Metal Levels and Renal Function in Korean Adults. International journal of environmental research and public health. 2022;19(11).

3. Chung MC, Hsu HT, Mao YC, Wu CC, Ho CT, Liu CS, et al. Association and mediation analyses among multiple metals exposure, plasma folate, and community-based impaired estimated glomerular filtration rate in central Taiwan. Environmental health : a global access science source. 2022;21(1):44.

4. Tsai HJ, Hung CH, Wang CW, Tu HP, Li CH, Tsai CC, et al. Associations among Heavy Metals and Proteinuria and Chronic Kidney Disease. Diagnostics (Basel, Switzerland). 2021;11(2).

5. Tsai CC, Wu CL, Kor CT, Lian IB, Chang CH, Chang TH, et al. Prospective associations between environmental heavy metal exposure and renal outcomes in adults with chronic kidney disease. Nephrology (Carlton, Vic). 2018;23(9):830-6.

6. Hsu LI, Hsieh FI, Wang YH, Lai TS, Wu MM, Chen CJ, et al. Arsenic Exposure From Drinking Water and the Incidence of CKD in Low to Moderate Exposed Areas of Taiwan: A 14-Year Prospective Study. American journal of kidney diseases : the official journal of the National Kidney Foundation. 2017;70(6):787-97.

7. Evans M, Discacciati A, Quershi AR, Åkesson A, Elinder CG. End-stage renal disease after occupational lead exposure: 20 years of follow-up. Occupational and environmental medicine. 2017;74(6):396-401.

8. Zhou R, Xu Y, Shen J, Han L, Chen X, Feng X, et al. Urinary KIM-1: a novel biomarker for evaluation of occupational exposure to lead. Scientific reports. 2016;6:38930.

9. Zheng LY, Umans JG, Yeh F, Francesconi KA, Goessler W, Silbergeld EK, et al. The association of urine arsenic with prevalent and incident chronic kidney disease: evidence from the Strong Heart Study. Epidemiology (Cambridge, Mass). 2015;26(4):601-12.

10. Kim NH, Hyun YY, Lee KB, Chang Y, Ryu S, Oh KH, et al. Environmental heavy metal exposure and chronic kidney disease in the general population. Journal of Korean medical science. 2015;30(3):272-7.

11. Sommar JN, Svensson MK, Björ BM, Elmståhl SI, Hallmans G, Lundh T, et al. End-stage renal disease and low level exposure to lead, cadmium and mercury; a population-based, prospective nested case-referent study in Sweden. Environmental health : a global access science source. 2013;12:9.

12. Navas-Acien A, Tellez-Plaza M, Guallar E, Muntner P, Silbergeld E, Jaar B, et al. Blood cadmium and lead and chronic kidney disease in US adults: a joint analysis. American journal of epidemiology. 2009;170(9):1156-64.

13. Barregard L, Trachtenberg F, McKinlay S. Renal effects of dental amalgam in children: the New England children's amalgam trial. Environmental health perspectives. 2008;116(3):394-9.

14. Lin JL, Lin-Tan DT, Li YJ, Chen KH, Huang YL. Low-level environmental exposure to lead and progressive chronic kidney diseases. The American journal of medicine. 2006;119(8):707.e1-9.

15. Lin JL, Lin-Tan DT, Yu CC, Li YJ, Huang YY, Li KL. Environmental exposure to lead and progressive diabetic nephropathy in patients with type II diabetes. Kidney international. 2006;69(11):2049-56.

16. Yu CC, Lin JL, Lin-Tan DT. Environmental exposure to lead and progression of chronic renal diseases: a four-year prospective longitudinal study. J Am Soc Nephrol. 2004;15(4):1016-22.

17. Lin JL, Lin-Tan DT, Hsu KH, Yu CC. Environmental lead exposure and progression of chronic renal diseases in patients without diabetes. The New England journal of medicine. 2003;348(4):277-86.

18. Lin JL, Tan DT, Hsu KH, Yu CC. Environmental lead exposure and progressive renal insufficiency. Archives of internal medicine. 2001;161(2):264-71.

19. Lin JL, Ho HH, Yu CC. Chelation therapy for patients with elevated body lead burden and progressive renal insufficiency. A randomized, controlled trial. Annals of internal medicine. 1999;130(1):7-13.

20. Rutowski J, Moszczyński P, Bem S, Szewczyk A. [Usefulness of determining urinary markers of early renal damage for monitoring nephrotoxicity during occupational exposure to mercury vapors]. Medycyna pracy. 1998;49(2):129-35.

21. Boogaard PJ, Houtsma AT, Journée HL, Van Sittert NJ. Effects of exposure to elemental mercury on the nervous system and the kidneys of workers producing natural gas. Archives of environmental health. 1996;51(2):108-15.

22. Schärer K, Veits G, Brockhaus A, Ewers U. High lead content of deciduous teeth in chronic renal failure. Pediatric nephrology (Berlin, Germany). 1991;5(6):704-7.
